# Heme alters biofilm formation in *Mycobacterium abscessus*

**DOI:** 10.1128/spectrum.02415-24

**Published:** 2024-12-23

**Authors:** Hadia Aftab, Jessica Samudio, Grace Wang, Lily Le, Rajesh K. Soni, Rebecca K. Donegan

**Affiliations:** 1 Department of Chemistry, Barnard College, Columbia University, New York, New York, USA; 2 Proteomics and Macromolecular Crystallography Shared Resource, Herbert Irving Comprehensive Cancer Center, Columbia University, New York, New York, USA; The Pennsylvania State University, University Park, Pennsylvania, USA

**Keywords:** *Mycobacterium abscessus*, heme utilization, biofilm, heme homeostasis, heme as a nutrient

## Abstract

*Mycobacterium abscessus* (Mabs) is commonly found in the cystic fibrosis (CF) lung. During infection, Mabs can form biofilms in the lung which reduce both the ability of the immune response to clear infection and the effectiveness of antibiotic therapy. In the CF lung, heme and hemoglobin levels are increased and may provide both iron and heme to Mabs cells. In this work, we show that exogenous heme altered Mabs biofilm formation and measured the effects of exogenous heme on protein level and metabolism in Mabs. Our findings suggest that heme impacts iron homeostasis in Mabs and affects other aspects of its metabolism, highlighting the potential role of heme as a critical nutrient for Mabs growth and biofilm formation.

**IMPORTANCE:**

*Mycobacterium abscessus* (Mabs) is commonly found in the cystic fibrosis (CF) lung, where Mabs can form biofilms that can reduce the efficacy of antibiotics. During infection, the CF lung can have more than 10 times the extracellular heme than that of a healthy lung. We have found that extracellular heme can change the way Mabs cells grow and form biofilms, which may have implications for pathogenesis.

## INTRODUCTION

Infections from the opportunistic pathogens of the nontuberculous mycobacteria (NTM) increase each year, particularly in patients with underlying lung diseases such as cystic fibrosis (CF) and chronic obstructive pulmonary disease (1). In CF patients, nearly half of all NTM infections are due to the fast-growing mycobacterium, *Mycobacterium abscessus* (Mabs) (2, 3). Current treatment regimens for Mabs infections require multiple antibiotics and treatment times of 18 months or longer and have a cure rate of less than 50% (2, 4). Even with treatment, Mabs infections in CF patients are associated with poor disease outcomes and are considered to be exclusion criteria for lung transplantation (2, 3).

The ability of Mabs to form biofilms is an important aspect of their pathogenicity. Within biofilms, Mabs cells are surrounded by secreted extracellular polymeric substances (EPS) that protect the pathogens from environmental stress, antibiotic treatment, and the innate immune response (5). The formation of biofilms by Mabs in municipal water supplies (6, 7) and on hospital equipment (8) has been suggested as a potential source of infection. In the CF lung, where Mabs infections are notoriously difficult to treat (9), Mabs biofilms have been identified in the sputum of infected patients (5).

Within the CF lung, oxidative stress contributes to inflammation and increases the levels of extracellular heme and hemoglobin (Hb) (10). In pediatric CF patients, the levels of extracellular heme in sputum were found to be in the micromolar range at 10 times higher than healthy controls (10), and heme levels within the CF lung are correlated with worse disease outcomes (11). These increased levels of heme may provide an iron source, heme source, or both for invading bacteria, and multiple CF pathogens have been found to increase heme use as the infection progresses (12, 13). However, how extracellular heme or Hb may alter Mabs growth and biofilm formation is not known. In this work, we determined that exogenous heme altered the growth of Mabs biofilms by preventing the formation of a submerged film and promoting the growth of unattached aggregates. Proteomics and metabolomics analyses revealed that heme exposure decreased levels of proteins involved in mycobactin biosynthesis and uptake, while also affecting proteins and metabolites linked to sulfur metabolism, carbohydrate metabolism, and other pathways. Importantly, multiple effects on biofilm growth and metabolite secretion in heme supplementation were distinct from those seen with iron supplementation. Altogether, our findings reveal a unique nutritional role of heme for Mabs and support future research to better understand how Mabs and other NTM use heme during infection.

## RESULTS

### Heme levels in *Mycobacterium abscessus* are similar between shaking and standing conditions

**Fig 1:**
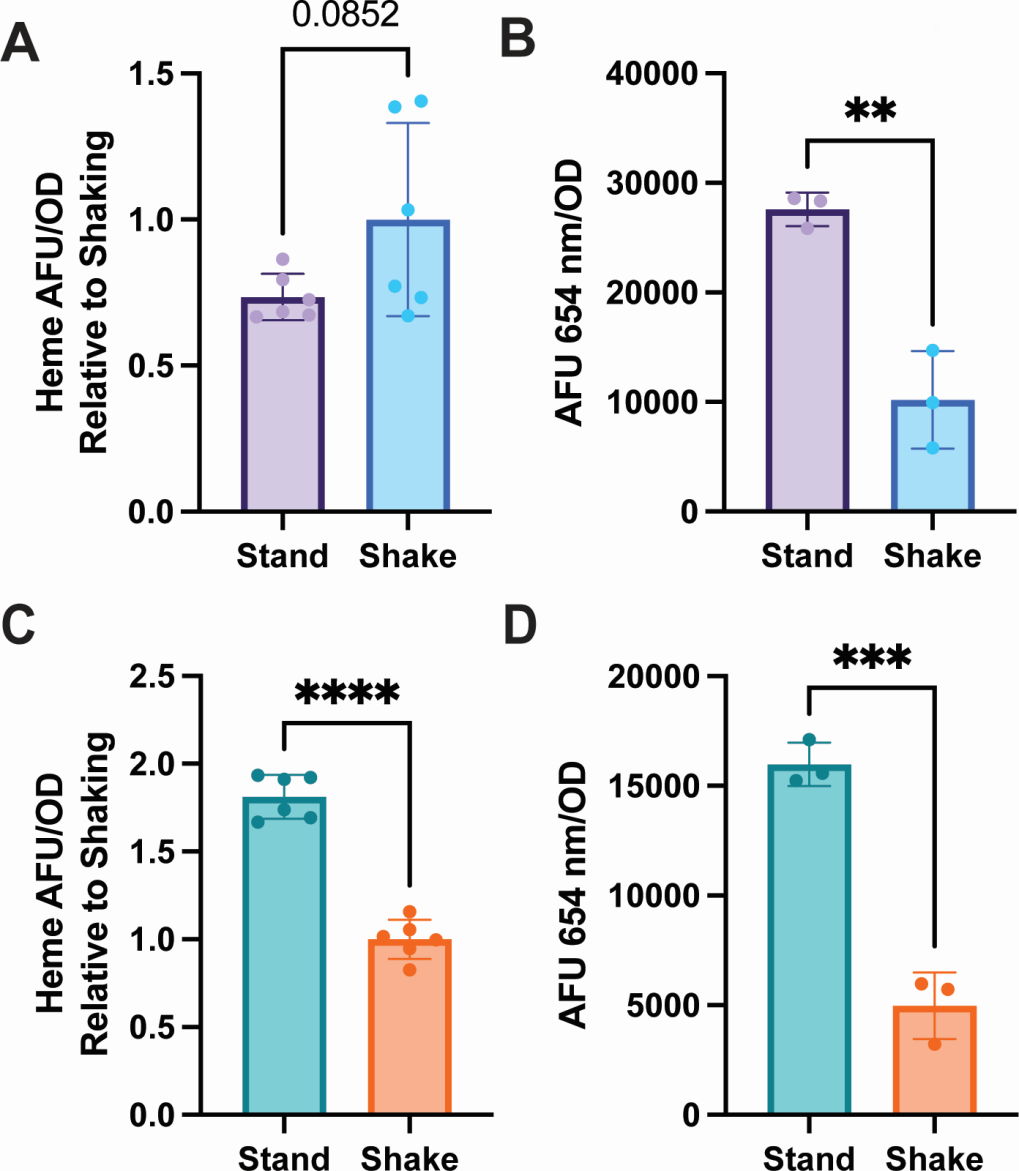
Effects of shaking and standing growth on intracellular heme and extracellular porphyrin in mycobacteria. (**A**) Intracellular heme arbitrary fluorescence units/optical density (AFU/OD) normalized to shaking in Mabs in standing conditions (stand) vs shaking (shake) at 37°C in Sauton’s media. (**B**) Extracellular porphyrin fluorescence (AFU 654 nm) normalized to OD in Mabs in standing conditions vs shaking at 37°C in Sauton’s media. (**C**) Intracellular heme AFU/OD normalized to shaking in Msm in standing conditions (stand) vs shaking (shake) at 37°C in Sauton’s media. (**D**) Extracellular porphyrin fluorescence normalized to OD in Msm in standing conditions vs shaking at 37°C in Sauton’s media. In all panels, statistical significance was measured by a two-tailed unpaired Student’s *t* test. *P* values calculated were *P* = 0.0852, and ** = 0.0030, **** < 0.0001, and *** = 0.0005, respectively. For A and C, data are normalized from two independent trials. For B and D, data are representative of two or more independent trials.

To determine heme levels in shaking and standing growth, the wild-type (WT) Mabs (ATCC 19977) cells were grown in Sauton’s media for 72 hours. Total intracellular heme and porphyrin secretion were measured for these cells via the porphyrin fluorescence method (14), and Mabs cell number was measured by optical denisty (OD_600_)to normalize for differences in growth. In Mabs, intracellular heme levels were not statistically different between standing and shaking conditions (Fig. 1A). However, Mabs cells grown in standing conditions secreted significantly more porphyrin into the media than cells grown in shaking conditions (Fig. 1B).

### Heme levels differ in *Mycobacterium smegmatis* between shaking and standing conditions

For comparison, we used the nonpathogenic fast-growing NTM, *Mycobacterium smegmatis* (Msm). WT Msm (ATCC mc^2^2155) cells were grown in Sauton’s media for 72 hours in identical conditions as Mabs cells. However, unlike Mabs, Msm cells grown in standing culture had more intracellular heme per cell than those grown in shaking culture (Fig. 1C). Additionally, we found that Msm-secreted porphyrin in media is higher in standing vs shaking cells (Fig. 1D). The secreted porphyrin from Msm was previously reported to be coproporphyrin III (copro) (15), and copro was verified to be the only detectable porphyrin in Msm media by the Iron and Heme Core at the University of Utah Center for Iron and Heme Disorders via ultra-performance liquid chromatography (data not shown).

To account for different reported cell numbers per OD for Mabs and Msm (16, 17) and the possibility of different cell sizes and volumes, we compared extracellular secreted porphyrin to intracellular porphyrin levels in Mabs and Msm. We found that in Mabs, the signal for secreted porphyrin was higher than intracellular porphyrin, whereas in Msm, the secreted porphyrin signal was lower compared to intracellular porphyrin (Fig. S1A). The purpose of porphyrin secretion by either Msm or Mabs is not currently known, though other bacteria secrete porphyrins to various effects (18–21).

### Exogenous heme reduces formation of submerged film by *Mycobacterium abscessus*

Msm and Mabs had different relative heme levels between standing and shaking conditions; therefore, we sought to determine if exogenous heme would alter the growth of Mabs or Msm cells as biofilms. Most bacteria are found as biofilms in nature, and biofilm formation via NTM is related to pathogenicity (21, 22). To measure biofilm formation, Msm and Mabs cells were grown in a modified 7H9 medium, without the addition of bovine serum albumin (BSA) or Tween-80 (7H9 in text), in 12-well and 96-well plates. 7H9 has ~150 µM iron and is considered a rich media and supports the growth of pellicles for both Mabs and Msm. For Msm and Mabs heme treatment, 5 and 25 µM heme were chosen to represent a range centered on the approximately 10 µM extracellular heme reported in juvenile CF sputum (10) and to be below the reported levels of heme used without any detriment to growth in previous Mabs (23) and Msm (24) experiments. In Mabs biofilms grown in 96-well plates, 5 µM heme added to 7H9 increased the growth of unattached cells (Fig. S1B) but reduced attached biofilm formation as measured by crystal violet (CV) assay (Fig. S1C). Growth in 12-well plates was observed as either a floating pellicle, a submerged film, or unattached aggregate growth. Mabs cells formed a thin pellicle that was increased by heme treatment (Fig. S1D) and had a thin film at the bottom of the well similar to what is seen in the Mabs 7H9 no glycerol photo (Fig. S1D). For Msm grown in 7H9 in 12-well plates, cells grew as a pellicle and formed a small film at the bottom of the well. Treatment of Msm with 5 µM heme had little effect on pellicle or film formation (Fig. S1E). In 96-well plates, Msm grown with 5 µM heme had no change in planktonic OD (Fig. S1F) and had decreased attached biofilm formation as measured via CV assay (Fig. S1G).

**Fig 2:**
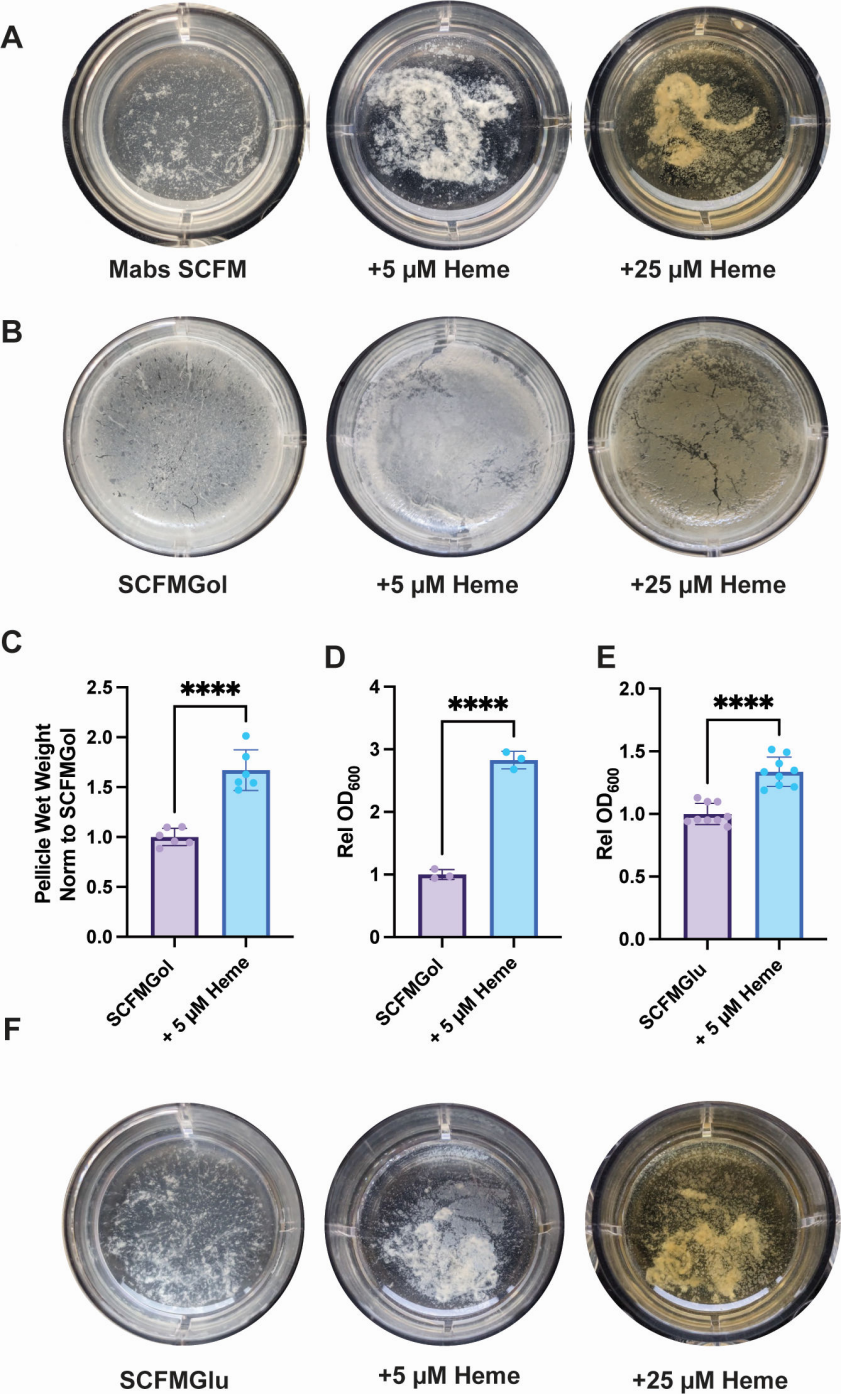
Effect of exogenous heme on biofilm formation in Mabs. (**A**) Representative images of Mabs pellicle growth in 12-well plates in SCFM media with 5 or 25 µM heme as indicated. (**B**) Representative images of Mabs pellicle growth in 12-well plates in SCFMGol media with 5 or 25 µM heme as indicated. (**C**) Effect of 5 µM heme treatment on Mabs pellicle formation in SCFMGol measured by wet weight. Data shown are normalized to untreated. (**D**) Effect of 5 µM heme treatment on Mabs film and unattached aggregate cell growth in SCFMGol measured by OD_600_. Data shown are normalized to untreated. (**E**) Effect of 5 µM heme treatment on Mabs film and unattached aggregate cell growth in SCFMGlu measured by OD_600_. Data shown are normalized to untreated. (**F**) Representative images of Mabs pellicle growth in 12-well plates in SCFMGlu media with 5 or 25 µM heme as indicated. Images in (**A, B, and F**) are representative of ≥3 wells per trial and ≥2 independent trials. Data in (**C and E**) are normalized from two independent trials, and data in (**D**) are representative of two independent trials. For (**C–E**), statistical significance was measured by a two-tailed unpaired Student’s *t* test, ****P < 0.0001.

It has been previously reported that a clinical isolate of Mabs cells (NJH12) formed submerged films attached to the bottom surface of wells when grown in a modified synthetic CF media (SCFM) (25) that is formulated to be nutritionally similar to CF sputum (26, 27). The ATCC 19977 strain of Mabs grown in this modified SCFM (referred to in the text as SCFM, see Materials and Methods) formed a similar submerged film (Fig. 2A). To test the effect of exogenous heme on submerged biofilm formation, we added 5 and 25 µM heme during growth. The addition of 5 µM heme reduced the formation of the attached film but increased the growth of unattached aggregates and supported the formation of a very small pellicle at the air-liquid interface (Fig. 2A). In SCFM, the addition of 25 µM heme reduced film formation, supported the growth of unattached aggregates, and increased the formation of a pellicle (Fig. 2A).

### Glycerol promotes pellicle formation in *Mycobacterium abscessus*

Mabs cells grown as biofilms in Sauton’s minimal media were previously shown to form a pellicle (25, 28). Both 7H9 and Sauton’s have glycerol added in the media, and we found that if glycerol was omitted from 7H9, we did not see pellicle formation via Mabs after 5 days of growth (Fig. S1D). Consequently, we tested Mabs biofilm formation in modified SCFM with glycerol added but no glucose or 1,2-dioleoyl-sn-glycero-3-phosphocholine (DOPC) (SCFMGol). The addition of glycerol in SCFMGol supported the formation of a pellicle in untreated and heme-treated Mabs (Fig. 2B). While both the untreated and heme-treated Mabs formed pellicles in SCFMGol, the morphologies of these pellicles were different. In heme treatment, pellicles were thicker and smooth, whereas in untreated Mabs, pellicles appeared thin and showed the wrinkling that is typical for mature mycobacterial pellicles (Fig. 2B) (29). The addition of heme increased pellicle formation measured by wet weight (Fig. 2C), and heme-treated cells had increased growth of non-pellicle cells measured by OD (Fig. 2D).

We also tested the effect of heme on Mabs biofilm formation in a simplified SCFM with glucose but without DOPC (SCFMGlu). In SCFMGlu, heme also supported the growth of Mabs non-pellicle cells (Fig. 2E). Mabs biofilm growth in SCFMGlu was similar to SCFM, as untreated Mabs formed a film attached to the bottom of the well (Fig. 2F). In SCFMGlu, no pellicle was formed by 5 days (Fig. 2F), but heme-treated Mabs cells (5 or 25 µm) grew as unattached aggregates instead of submerged film and formed a small pellicle (Fig. 2F).

### Exogenous heme alters protein expression in *Mycobacterium abscessus*

To identify proteins or pathways in Mabs cells grown in SCFM that are altered by heme treatment, we used label-free quantitative (LFQ) proteomics (Prof. Rajesh Soni, CUIMC Proteomics Core) to identify protein level changes in heme-treated cells. Cells were grown in SCFM with or without 25 µM heme for 48 hours. Cells were pelleted, including any cells growing as a pellicle or film, washed with water, and frozen for analysis. Proteins with greater than a twofold decrease in treated relative to untreated cells and a *P*-value < 0.05 are reported in Table S1 (Table S1). Proteins with greater than a twofold increase in treated relative to untreated cells and a *P*-value < 0.05 are reported in Table S2 (Table S2).

There were 12 proteins identified as having more than a twofold decrease in heme treatment relative to SCFM and an additional 9 were only detected in Mabs cells grown in SCFM (Table S1). Of these 21 proteins, 13 are annotated as iron homeostasis proteins and are listed in Table 1. These proteins are either part of the synthesis or uptake pathways of the iron-scavenging siderophore mycobactin (23) or part of the ESX-3 secretion system which is necessary for iron and zinc homeostasis in mycobacteria and is required for mycobactin utilization (23, 30).

**TABLE 1:** ESX-3 or mycobactin synthesis and uptake proteins with significantly reduced levels in heme-treated cells relative to untreated

Protein function	Gene ID	Log2 fold change	*P* value^*a*^
Mycobactin synthesis (MbtF)	MAB_2123	−5.3	0.0124
ESX-3 (eccB3)	MAB_2233c	−4.7	0.0018
ESX-3	MAB_2234c	−4.4	0.0001
ESX-3	MAB_2226c	−3.2	0.0137
ESX-3	MAB_2232c	−2.9	0.0011
ESX-3	MAB_2227c	−2.5	0.0104
Mycobactin synthesis (MbtB)	MAB_2121c	−2.4	0.0067
Mycobactin uptake (IrtB)	MAB_2261c	−1.7	0.0001
ESX-3	MAB_2229c	−1.4	0.0197
**Iron homeostasis proteins with measurable expression in SCFM but not SCFM + Heme** ^***b***^			
Mycobactin uptake (IrtA)	MAB_2262c	NA	NA
ESX-3	MAB_2225c	NA	NA
Mycobactin synthesis (MbtC)	MAB_2120c	NA	NA
Mycobactin synthesis (MbtI)	MAB_2245	N/A	N/A

^*a*^ *P*-values were calculated using multiple *t*-tests. Twenty-one total proteins were identified that had greater than twofold change in expression and a *P*-value < 0.05.

^*b*^ An additional 18 proteins were identified as having measurable levels only in SCFM or only in SCFM + H. Protein identities were determined after cutoff (see Tables S1 and S2). Fold change and p-values listed as N/A were not calculated as these proteins were not detcted in heme treated samples.

A total of 18 proteins were either increased greater than equal twofold in heme treatment relative to untreated or measured only in heme-treated cells (Table S2). Many of these proteins have not been characterized, and further work is needed to determine their function in Mabs during growth in exogenous heme. One protein of interest that was increased ~2.3× in heme-treated cells was MAB_4003c (Table S2). Putatively, this protein is a uridine diphosphate (UDP)-glucose 4-epimerase GalE1, which interconverts UDP-glucose with UDP-galactose. UDP-glucose is a precursor for cellulose biosynthesis but is also an intermediate in a multitude of other pathways including those of starch and sugar metabolism and nucleotide metabolism.

### Effect of iron supplementation on *Mycobacterium abscessus* pellicle formation

**Fig 3:**
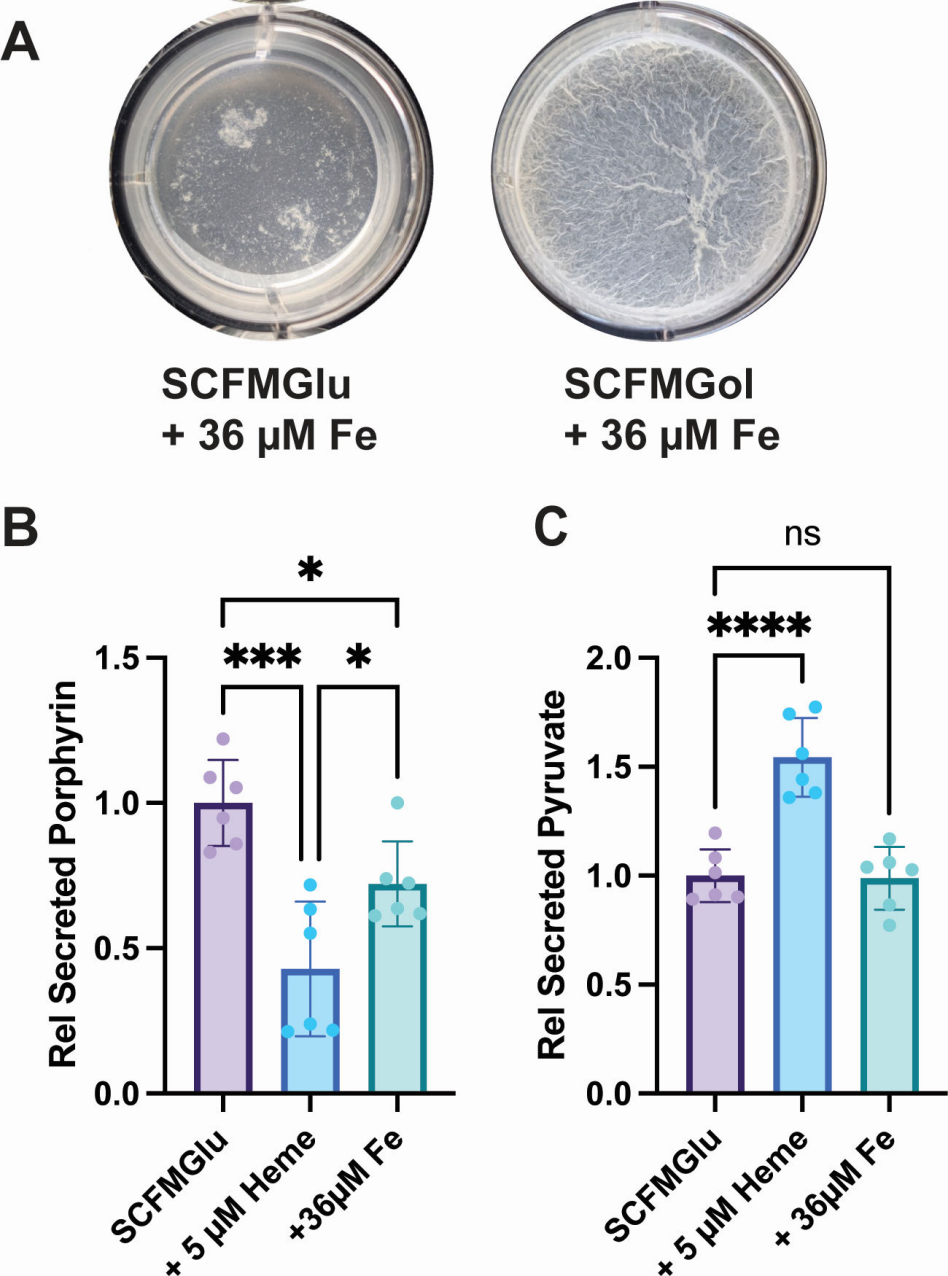
Heme and iron have unique effects on Mabs biofilm formation and metabolite secretion. (**A**) Representative images of biofilm growth with iron supplementation in SCFMGlu and SCFMGol. (**B**) Porphyrin secretion by Mabs in SCFMGlu treated with 5 µM heme or 36 µM iron (Fe) relative to untreated (SCFMGlu). (**C**) Pyruvate secretion by Mabs in SCFMGlu treated with 5 µM heme or 36 µM iron (Fe) relative to untreated (SCFMGlu). Images in (**A**) are representative of three wells per trial and two independent trials. Statistical analysis in (**B**) was assessed by one-way analysis of variance (ANOVA) with Tukey’s multiple comparisons test. Calculated *P* values were ***P = 0.0002, *P = 0.0431, and *P = 0.0325, respectively. Statistical significance in (**C**) was assessed by one-way ANOVA with Dunnett’s post-hoc test using untreated Mabs in SCFMGlu as the reference. Calculated *P* values were ****P < 0.0001 and ns = 0.9882. Data in (**B**) and (**C**) are normalized between two independent trials.

Previous studies have shown that the addition of iron promotes pellicle formation in Msm (31), and heme can be used by mycobacteria as an iron source (23, 32, 33). Our additional finding that Mabs treated with heme had a reduction in mycobactin biosynthesis, and uptake proteins led us to examine if iron addition could account for the changes in biofilm formation seen in Mabs with heme treatment. The addition of 36 µM iron, added as ferrous sulfate heptahydrate, to SCFMGlu led to submerged film formation (Fig. 3A) similar to untreated SCFMGlu (Fig. 2F). The addition of iron in SCFMGlu increased cell growth compared to untreated cells, but cells treated with 5 µM heme grew better in these conditions (Fig. S2A). In SCFMGol, the addition of iron increased pellicle formation as measured by wet weight (Fig. S2B), but 5 µM heme-treated cells had more pellicle by weight. The appearance of the iron-treated pellicle was thin and wrinkled (Fig. 3A) like that of untreated Mabs (Fig. 2B).

### Heme alters metabolite secretion by *Mycobacterium abscessus*

During biofilm growth, Mabs secretes porphyrin into the culture media in both 7H9 and SCFM, as in shaking and standing cultures in Sauton’s media reported above (Fig. 1B). The addition of 5 µM heme reduced the secretion of porphyrin by ~50% in SCFMGlu (Fig. 3B) and SCFMGol (Fig. S2C, extra) but not intracellular porphyrin (Fig. S2C, intra). This reduction in porphyrin fluorescence is not due to inner filter effects of heme (Fig. S2D). SCFM has ~3.6 µm iron added in formulation, and the addition of 36 µM iron to SCFMGlu or SCFMGol did not reduce porphyrin secretion to the same level as heme (Fig. 3B; Fig. S2C). Added iron did increase intracellular heme levels, suggesting that the iron was taken up and utilized by Mabs (Fig. S2E).

Porphyrin and heme biosynthesis in mycobacteria require glutamate, which is a central metabolite in cellular metabolism. The addition of heme reduced porphyrin biosynthesis as levels of secreted porphyrin were reduced. We determined that during biofilm growth in SCFMGlu, Mabs secreted pyruvate which is common for bacteria in amino acid replete media such as SCFM (Fig. 3C) (34). We hypothesized that the addition of exogenous heme could increase pyruvate secretion as the cataplerotic pull of heme biosynthesis would be reduced when heme was taken up. Indeed, the addition of 5 µM heme increased pyruvate secretion by ~1.5-fold (Fig. 3C); however, the addition of 36 µM iron did not increase pyruvate secretion in these conditions (Fig. 3C).

### *Mycobacterium abscessus* aggregate cells had reduced eDNA and cellulose levels and associated with heme

**Fig 4:**
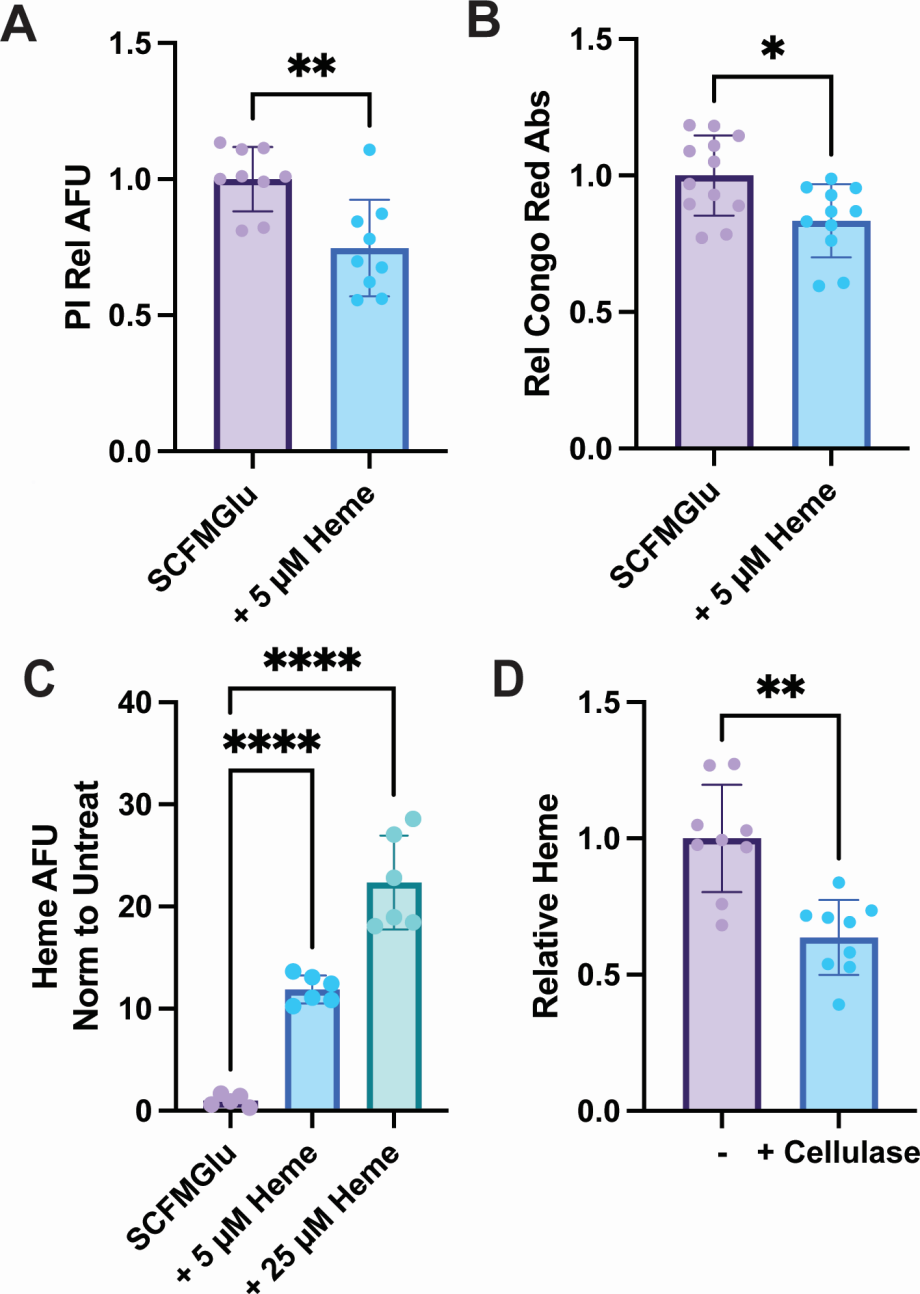
Heme reduces PI and CR staining of Mabs cells. (**A**) eDNA measured by PI fluorescence and (**B**) cellulose measured by CR absorbance in Mabs submerged films and unattached aggregate cells grown in SCFMGlu in 12-well plates for 5 days. (**C**) Mabs cells grown in SCFMGlu were treated with 5 or 25 µM heme for 1 hour, and total heme was measured. (**D**) Total heme measurement of cells grown in various SCFM formulations + 25 µM heme and treated with water (−) or 5 mg/mL cellulase in water (+ cellulase) for 30 minutes. Statistical significance in (**A and B**) was measured by a two-tailed unpaired Student’s *t* test. *P* values calculated were **P = 0.0025 and *P = 0.0105, respectively. Statistical analysis in (**C**) was assessed by one-way analysis of variance (ANOVA) with Dunnett’s post-hoc test using untreated Mabs in SCFMGlu as the reference. Calculated *P* values were <0.0001. For (**D**), a two-tailed paired Student’s *t* test was used, and the calculated **P value = 0.0018. Data in (**A, B, and C**) are normalized for two independent trials of ≥3 wells per trial. Data in (**D**) are from three wells each of three separate SCFM formulations with glucose, glycerol, or both.

Mabs cells treated with heme had reduced surface attached film but increased growth as unattached aggregates in SCFM. This suggests that heme treatment altered how Mabs cells attached to the surface or interacted with each other. It has been previously shown that extracellular DNA (eDNA) and cellulose are important structural components for Mabs submerged film attachment (25), and we found that DNase and cellulase disrupted unattached aggregates formed by heme treatment (Fig. S3A). We measured eDNA and cellulose in Mabs cells grown as biofilms in SCFMGlu where untreated cells grew mostly as submerged film, and 5 µM heme-treated cells grew mostly as unattached aggregates (Fig. 2F). Film and aggregate cell eDNA were measured using propidium iodide (PI) fluorescence. Compared to untreated cells, heme-treated cells had ~25% less PI fluorescence (Fig. 4A). Cellulose was measured using the cellulose binding dye Congo red (CR), and heme treatment significantly reduced CR binding of Mabs cells by ~15% (Fig. 4B). Mabs biofilms grown in SCFM ±25 µM heme were stained for total biofilm using acridine orange (AO), which binds both intracellular and eDNA and for cellulose using the cellulose binding dye calcofluor white (CW). Both untreated and heme-treated films had AO and CW staining (Fig. S3B); however, quantification of CW relative to AO (CW/AO) showed reduced staining of cellulose by CW staining in heme-treated biofilms (Fig. S3C).

We observed that Mabs cell aggregates grown in heme treatment were brown in color when pelleted and washed (Fig. S3D), and SCFMGol pellicles appeared brown in color when grown in 25 µM heme (Fig. 2B). To measure heme binding or heme association with the aggregate cells, Mabs cells were grown in SCFMGlu and then treated with 5 or 25 µM heme for 1 hour. Cells were pelleted and washed, and total heme was measured for untreated and treated cells. While total heme would measure both intracellular heme and any heme attached to the cells, the comparison with treated and untreated cells at a short time point of heme treatment should provide an approximation for heme that is associated with the cells or taken up by the cells. In 5 µM heme-treated cells, total heme increased ~11-fold, while in cells treated with 25 µM heme, the total heme increased ~22-fold compared to untreated (Fig. 4C). Finally, we measured heme levels in cells grown in 25 µM heme, pelleted, washed, and then treated with 5 mg/mL cellulase in water for 30 minutes. Heme associated with the cells was measured by a modified total heme assay. Cells treated with cellulase had ~30% less heme than cells that were treated with water instead of cellulase (Fig. 4D).

### Heme alters *Mycobacterium abscessus* metabolism

Given that heme altered porphyrin and pyruvate secretion in Mabs and reduced staining of eDNA and cellulose, we wanted to determine if there were metabolic changes in Mabs due to exogenous heme treatment. Mabs cells were grown in SCFMGlu ±5 µM heme for 5 days, pelleted, washed, and frozen at the same OD for untargeted metabolomics performed by Creative Proteomics (Shirley, New York). Metabolites were measured and identified by Creative Proteomics as previously described (35, 36). Multiple metabolites were identified in both the positive and negative mode. Metaboanalyst (37) was used to determine enrichment via quantitative enrichment analysis with the Kyoto Enclyopedia of Genes and Genomes (KEGG) metabolite library for both the positive and negative mode data set of identified metabolites. The top-enriched metabolite sets are shown for both positive and negative mode (Fig. S4). Twenty-two metabolite sets were identified as significantly altered in heme treatment, including porphyrin synthesis as the heme synthesis precursor coproporphyrin III, identified as coproporphyrin, was measured to be reduced in heme treatment (Fig. S5). Other metabolites identified as enriched were then filtered to remove those sets which included metabolites not likely synthesized in mycobacteria such as alpha-linoleic acid and S-lactoylglutathione. These metabolites are likely misidentified due to the use of general metabolite databases. Metabolite sets identified as enriched with multiple metabolites or with a metabolite and an enzyme identified as significantly altered by heme via proteomics are discussed briefly below and reported in Fig. S6 to S11 (Fig. S6 to S11).

Exogenous heme altered sulfur and methionine metabolism in Mabs as heme treatment increased levels of metabolites associated with methionine salvaging while decreasing thiosulfate levels (Fig. S6). A putative sulfite reductase (MAB_2492) was below detectable levels in heme-treated cells (Table S1). In addition, multiple metabolites involved in purine metabolism were altered as heme treatment reduced levels of metabolites associated with purine catabolism and significantly increased levels of adenosine-monophosphate relative to SCFMGlu (Fig. S7). Flavin metabolism was enriched with heme treatment as both flavin adenine dinucleotide and flavin mononucleotide (FMN) were increased in heme-treated Mabs relative to SCFMGlu (Fig. S8). The FMN derivative dimethyl benzimidazole, which is used in the synthesis of the lower ligand of cobalamin (vitamin B12), is also increased in heme treatment (Fig. S8). Tryptophan metabolism (Fig. S9) and glycerophospholipid metabolism (Fig. S10) were also identified as altered in heme treatment. Finally, three metabolite sets were identified as enriched that included UDP-glucose as one of the metabolites (Fig. S4 and S11). UDP-glucose is a substrate of UDP-glucose 4-epimerase GalE1 (Table S2) which was increased in heme treatment.

### The effect of hemoglobin on *Mycobacterium abscessus* biofilms

Hemoglobin-bound heme is the most abundant source of heme for pathogens during infection (38). Consequently, we tested the effect of both bovine Hb (BovHb) and human Hb (HuHb) on Mabs biofilm formation. The addition of 6 µM BovHb supported the formation of a submerged film, aggregated unattached cell growth, and a pellicle by Mabs in SCFM media (Fig. S12A). The addition of 3 µM HuHb supported the formation of a submerged film and pellicle; however, HuHb forms visible aggregates in SCFM at 37°C as Mabs cells grow (Fig. S12A, arrow). This aggregation is accelerated when Mabs cells are present, though if this is a result of the Mabs cells, it is yet to be determined. The addition of human serum albumin and heme (HSA-heme, Fig. S12B and C), equine apo-myoglobin (Apo-Mb), or equine myoglobin (Mb) does not support attached biofilm formation unlike Hb (Fig. S12D and E). Finally, by microscopy, we see that HuHb colocalizes with the Mabs biofilm (AO) and cellulose (CW; Fig. S12F).

## DISCUSSION

Our study on the effects of exogenous heme on biofilm formation in Mabs adds to the growing work on heme utilization in mycobacteria (24, 32, 33, 39). Since heme iron is the most abundant iron source in the human host (38), pathogenic mycobacteria may have evolved to use heme during infection as has been seen in other opportunistic lung pathogens (12, 13, 40). Previous work has demonstrated that exogenous heme may be used as an iron source in other mycobacteria (32, 33, 39, 41) and in Mabs (23), and exogenous heme may be used directly in hemoproteins by mycobacteria (24). We have found that exogenous heme reduced siderophore production and uptake and appeared to be a better-utilized nutrient for Mabs growth in SCFM than supplementation with iron salts. In addition, exogenous heme altered Mabs biofilm formation by supporting pellicle growth and reducing attached film formation. Finally, we found that heme supplementation provided nutrition beyond iron to Mabs altering metabolite secretion, and exogenous heme altered multiple metabolic pathways in Mabs.

Heme treatment of Mabs cells grown in the low iron media SCFM resulted in a decrease of proteins associated with mycobactin synthesis and uptake via the ESX-3 complex (Table 1). Previous work in Mabs has also found that heme supplementation can rescue the reduced growth of a Mabs strain lacking the mycobactin synthesis gene *mbtE,* thus overcoming iron restriction due to limited iron uptake (23). Our findings suggest that exogenous heme is taken up by Mabs in low-iron environments and used as an iron source, reducing the need for siderophore-dependent uptake even with low micromolar levels of iron available. In addition, 5 µM heme increased Mabs growth more than supplementation with 36 µM iron in both SCFMGlu as non-pellicle cell growth (Fig. S2A) and as pellicle growth in SCFMGol (Fig. S2B). Together, our findings indicate that regulation of mycobactin-dependent iron uptake and heme uptake is coordinated in Mabs, and heme provides better nutrition for Mabs than iron added as iron salts. Though further studies are necessary to determine if heme is a preferential source for iron in Mabs in other conditions and to identify the mechanisms by which heme uptake is regulated.

Heme treatment also altered Mabs biofilm formation in at least two ways. First, heme reduced the formation of a submerged film and promoted the growth of unattached aggregate cells (Fig. 2). This effect was limited to heme, as iron and Hb supplementation did not reduce submerged film formation (Fig. 3; Fig. S12). The lack of surface attachment of heme-treated Mabs cells and the association of heme with cells and EPS could be an artifact of the sticky nature of heme when not protein bound. To this end, heme associated with Mabs cells and likely altered surface attachment via eDNA and/or cellulose as levels of both are decreased in heme treatment (Fig. 4). In addition, the disruption of cellulose in Mabs aggregates led to a reduction in heme associated with the cells (Fig. 4D), suggesting that either heme was bound directly to cellulose or that the structural aspects of cellulose in the EPS were involved in heme association with the aggregates.

How the addition of heme may alter the levels of cellulose and eDNA in the EPS is not known; however, multiple metabolites associated with the synthesis of DNA and cellulose were altered in heme treatment. First, metabolites associated with purine catabolism were reduced, while adenosine monophosphate was increased in heme treatment (Fig. S7). Second, levels of both UDP-glucose, the disaccharide maltose, and the putative enzyme UDP-glucose 4-epimerase GalE1 (Table S2, MAB_4003c) were increased in heme treatment. UDP-glucose is a metabolite in multiple pathways including cellulose biosynthesis, carbohydrate metabolism, and nucleotide sugar metabolism and is a likely substrate of MAB_4003c. However, given that these sugars could be in multiple isomeric forms with the same molecular weight, it is possible that these are instead other sugars. For example, the metabolite could be UDP-galactose instead of UDP-glucose, and maltose could be some other di-hexose with the same molecular weight. While further studies are needed, it is plausible that the effects of heme supplementation on these pathways could result in the altered levels of eDNA and cellulose in the EPS of Mabs biofilms.

The second way in which heme treatment of Mabs cells altered biofilm formation was by increasing the formation of a pellicle at the air-liquid interface. In the pellicle-forming media SCFMGol, iron supplemented Mabs formed a pellicle that is wrinkled and thin (Fig. 3A). Heme-treated cells, however, formed a pellicle that is thicker and has a smoother appearance (Fig. 2B). These distinct pellicle morphologies are similar to pellicles formed by the two distinct Mabs morphotypes termed smooth and rough (42). The two morphotypes are distinguished by the appearance of their colonies on agar plates where the smooth morphotype is characterized by smooth wet colonies on agar plates and produces cell surface glycopeptidolipids (GPLs) (43), and the rough colony morphotype is characterized by wrinkled dry colonies on agar plates, lacks GPLs, grows with a cording phenotype (43), and has been proposed to be more virulent (44). Both morphotypes produced pellicle biofilms but with distinct morphologies (42). Smooth morphotypes produced a thick pellicle, described by the authors as “oleaginous,” (42) and looked similar to the smooth thick pellicles formed by heme-treated cells (Fig. 2B). Rough morphotypes produced a thinner wrinkled pellicle termed “waxy” by the authors (42) that was more similar to the wrinkled pellicles formed in iron treatment (Fig. 3A). Given that the appearance of the pellicles is due to the surface properties of the GPLs and since heme associates with the Mabs cells and EPS components, it is possible that heme association altered Mabs surface interactions within the pellicle leading to a smoother appearance. However, much more work is needed to determine exactly how heme and iron alter Mabs pellicle morphology.

Heme is often considered as an alternative iron source for pathogenic bacteria, but our findings also suggest a role for exogenous heme in altering carbon metabolism in Mabs. The addition of heme in SCFMGlu reduced the secretion of porphyrin and increased the secretion of pyruvate (Fig. 3B and C). Given that porphyrin synthesis in Mabs requires glutamate (45), it is possible that exogenous heme reduces *de novo* heme biosynthesis, and in turn porphyrin biosynthesis, which reduces the cataplerotic pull of glutamate from central carbon metabolism. Intriguingly, the addition of iron reduced porphyrin secretion (Fig. 3B), although to a lesser extent than heme, and increased intracellular heme levels (Fig. S2E). This suggests that Mabs cells in SCFMGlu are somewhat iron and heme deficient, and the addition of iron salts in the media increases iron uptake and use. Supplementation with 36 µM Fe, however, had no effect on pyruvate secretion (Fig. 3C), supporting an additional role for exogenous heme as a nutrient for Mabs beyond that as solely an iron source.

The addition of exogenous heme also influenced multiple metabolic pathways in Mabs. Given that the effect of exogenous heme on mycobacterial metabolism is not well understood, these findings provide a basis for future research. The top pathways that were enriched included glycerophospholipid metabolism, porphyrin metabolism, riboflavin metabolism, sulfur metabolism, and methionine metabolism (Fig. S4). Many of these pathways are important for maintaining redox balance within cells. Of particular interest for future studies are the changes in sulfur and methionine metabolism in Mabs grown with exogenous heme (Fig. S6). Heme treatment increased metabolites associated with methionine salvage despite having methionine supplied to cells in the SCFMGlu media. Whether this is due to the need for these metabolites in other pathways, such as terpenoid biosynthesis, or if heme treatment increases the need for methionine or S-adenosylmethionine in Mabs remains to be determined. Heme treatment also significantly decreased levels of putative sulfite reductase (MAB_2492, Table S1) and thiosulfate levels (Fig. S6). The effect of exogenous heme on these pathways in Mabs warrants further study as methionine synthesis is necessary for *Mycobacterium tuberculosis* to establish an infection in mice (46), suggesting that targeting these pathways may present a viable strategy to combat mycobacterial infections.

Growth of Mabs cells in SCFM with either BovHb or HuHb supported both film and pellicle formation. In 96 well plates, we saw an increase in OD and an increase in attached biofilm measured by CV. The increase in unattached cell growth was also seen in HSA-heme, Apo-Mb, and Mb treatment; however, the increase in both unattached cell growth and biofilm was only seen for Hb-treated cells. HuHb also forms aggregates in SCFM over time, and these aggregates appear to be incorporated into the submerged films formed by Mabs. These findings support the dual role of heme in altering biofilm formation, in which protein bound heme altered growth and pellicle formation, but “free” heme alters interactions between Mabs cells and each other or with surfaces.

These results support a role for heme as both an iron source in low-iron media and as a nutrient for Mabs. Heme and Hb increase Mabs growth and alter biofilm formation in a media designed to mimic the nutritional environment of the CF lung providing support for the consideration of heme and hemoglobin in future studies of Mabs growth and pathogenesis.

## MATERIALS AND METHODS

### Mycobacterial cell culture

Msm (ATCC mc^2^2155) and Mabs (ATCC 19977) were purchased from ATCC and grown from lyophilized cells in Difco 7H9 media supplemented with albumin, dextrose, salt (ADS) and Tween-80. Five milliliter glycerol per liter was used in all formulations of 7H9 in this work. Cells were grown for 2 days, and glycerol stocks were made and stored at −80°C for use. For assays, Msm or Mabs cells were grown from cell stocks in 7H9 with ADS for 2 days. For culture in Sauton’s, SCFM, or 7H9 media, cells were pelleted, washed, and resuspended in the appropriate culture media to remove residual BSA and Tween-80. Cells were diluted in culture media at 0.05–0.1 ODs, unless otherwise noted. Heme used in cell culture was hemin chloride (Sigma) dissolved in 0.1 M NaOH, and heme concentration was determined by absorbance at 612 nm using the extinction coefficient of 4431 M^−1^ cm^−1^ (47). The Hb stocks were made by dissolving bovine Hb (Sigma) or human Hb (Sigma) in water and sterile filtering. Stocks were made fresh before use, and the concentration was based on measured weight of Hb per milliliter water.

### SCFM and 7H9 media

SCFM media was modified from SCFM2 described in Turner et al. (26) without DNA or Mucin as has been previously used to study Mabs biofilms (25). For SCFMGlu, no DOPC was added. For SCFMGol, no glucose or DOPC was added, but 5 mL/L glycerol was added. For 7H9, the media was made as recommended by the manufacturer with some modifications. For initial growth from cell stocks, 7H9 media was added at 4.7 g/L along with 5 mL/L glycerol, 2 g/L glucose, 0.85 g/L NaCl, 5 g/L BSA, and 0.05% Tween-80 (7H9 + ADS). For the growth of Mabs as biofilms, 7H9 without added BSA or Tween-80 was used.

### Shaking vs standing growth

For the comparison of shaking vs standing growth, cells were grown from stocks as above. For both shaking and standing culture, 2 mL of cells was grown in 14 mL sterile round bottom culture tubes with caps loose. Cells were started at 0.05 OD for shaking culture and 0.5 OD for standing culture in Sauton’s media with an additional 0.05% Tween-80 added to the base media to prevent aggregation (Hi-Media Sauton’s Fluid Medium Base). Cells were grown for 2 days at 37°C.

### Biofilm growth

Mabs or Msm cells were grown as biofilms in 12-well Corning Costar Clear Plates or Falcon 96-Well, Non-Treated, U-Shaped-Bottom microplates. Cells were grown from glycerol stocks in 7H9 media with BSA and Tween-80 for 2 days shaking at 37°C to reach saturated growth. Cells were pelleted, washed, and resuspended in the media for biofilm growth. Cells were diluted to 0.05 OD in media in plates. In 12-well plates, 2 mL/well was added, in 96-well plates, 200 µL/well was added. For 12-well plates, plates were sealed with parafilm to reduce evaporations and placed in a 37°C standing incubator. In the 96-well plate, the outer wells were filled with water, and the plate was sealed with parafilm to reduce evaporation. Beakers with water were kept in the standing incubator to create a humid environment and to reduce evaporation. Biofilm formation was monitored over 5–7 days.

### Pellicle weight measurements

Mabs pellicle cells were removed from liquid surface by scraping with a clean metal scoop and transferring to a pre-weighed microcentrifuge tube. Pellicles were centrifuged at 12,000 × *g* for 5 minutes, and liquid was removed. This was repeated, and pellicle weight was measured.

### Crystal violet assay

Attached biofilms were measured via a CV assay (48). Briefly, unattached cells and media were removed via pipetting, and the OD was measured as planktonic cells in Falcon 96-well Clear Flat Bottom TC-treated Culture Microplates using absorbance at 600 nm on a Tecan Spark multimode microplate reader. Cell media with or without heme or Hb was used as a background measurement for OD. Wells in the growth plate were rinsed with water 2×, and 0.1% CV dye in water was added for ~20 minutes. Two hundred microliter CV was added to 96-well plates. Two milliliter CV added to 12-well plates to measure total attached biofilm. To measure only submerged film in 12-well plates, 500 µL CV was added. To measure submerged film and pellicle in 12-well plates, 2 mL CV was added. After incubation, CV was removed, and wells were washed 3× with water and allowed to dry inverted. Eighty percent ethanol in water was added at the same volume as used for CV and incubated at room temperature (RT) for 30 minutes to extract CV from stained biofilms. Two hundred microliter of the extracted CV was transferred to a clear flat bottom 96-well plate, and absorbance at 550 nm was measured.

### Propidium iodide assay

For the propidium iodide assay, an equal cell number as determined by OD was pelleted and washed with water and resuspended in phosphate buffered saline (PBS). Two hundred microliter of cells in PBS was plated into a black 96-well Greiner FLUOTRAC plate. Twenty microliter PI was added from a 10× stock of 5 µM PI solution diluted from 1 mg/mL PI in water purchased from Sigma. Fluorescence was measured using a Tecan Spark microplate reader with excitation at 535 nm and emission spectra read from 580 to 700 nm. Emission peak at 630 nm was used.

### Congo red assay

For the Congo red assay, equal cell number as determined by OD was pelleted and washed with water. One hundred microliter of 40 µg/mL CR in PBS was added, and cells were resuspended in CR solution and incubated at room temperature for ~30 minutes. Cells were pelleted and washed 3× with PBS to remove unbound CR, and CR was extracted using 150 µL 80% ethanol in water for 30 minutes at RT. Cells were pelleted, 100 µL supernatant was transferred to a clear flat bottom 96-well plate, and the absorbance spectra were measured from 400 to 600 nm. The peak absorbance value at 510 nm was used.

### Enzyme treatment of Mabs biofilms

Mabs cell aggregates were taken from heme-treated biofilms grown in 7H9 (without BSA or Tween) in 12-well plates for 5 days at 37°C. The aggregates were gently removed via pipetting to not disturb structure and placed in 500 µL water in a 12-well plate. CELLULYSIN Cellulase from Sigma was dissolved in water at 5 mg/mL. Wells were treated for 1 hour with 5 mg/mL cellulase (+ cellulase) or water (− cellulase) at 37°C. Fifty microliter DNase I – XT from NEB was mixed with 50 µL 10× buffer (supplied by NEB) and added to each well. One milligram of Proteinase K (20 mg/mL NEB) per well was added. Cells were incubated at 37°C for 1 hour, and the aggregates were imaged.

### Porphyrin fluorescence assays

The porphyrin fluorescence assay was used to measure both intracellular heme and extracellular porphyrin. The intracellular heme assay was done as previously described (14, 24). Briefly, cells were grown as described for each experiment, pelleted, washed, and put at the same OD. Cells were treated overnight in 500 µL of 20 mM oxalic acid. The next day, 500 µL of 2 M oxalic acid was added, mixed, and split into two tubes for each sample. One set of sample tubes was boiled for 30 minutes, and the other set was kept in the dark at room temperature. After boiling, all samples were spun down for 2 min at 9,000 × *g*. Two hundred microliter each of sample was added to a black 96-well Greiner FLUOTRAC plate. Fluorescence was measured using a Tecan Spark microplate reader with excitation at 400 nm and emission spectra read from 550 to 700 nm. The unboiled samples spectral signal was of intracellular non-heme porphyrin, and the boiled sample spectral signal was of total intracellular porphyrin, including heme. The intracellular heme fluorescence was calculated from the boiled minus unboiled signals using the peak at 662 nm. For secreted or extracellular porphyrin, 150 µL of cell culture media was taken and pelleted at 9,000 × g to remove any cells. One hundred microliter of cell-free media and 100 µL of 2 M oxalic acid were added to 96-well plates, and the spectra was measured as above. The extracellular porphyrin signal was calculated using the peak at 654 nm.

### Heme association assays

For the heme association assays, Mabs cells were grown from stocks in 7H9 complete media with BSA and Tween for 2 days. Cells were then pelleted to remove 7H9, resuspended in SCFMGlu and diluted 1:100 into three separate cultures in SCFMGlu media for 2 days. Cells were then treated with nothing, 5 or 25 µM heme for 1 hour in standing culture at 37°C to limit growth rate. Cells were pelleted, washed to remove unbound heme, and then resuspended in 500 µL of 20 mM oxalic acid overnight at 4°C. Total heme was then measured as described above via porphyrin fluorescence.

For cellulase treatment, cells were grown for 12-well biofilm assays in SCFM, SCFMGlu, and SCFMGol with 25 µM heme as described above for biofilm growth. The unattached aggregate cells were collected, washed to remove media and unbound heme, pelleted, and frozen for future analysis. Cell aggregate pellets were allowed to thaw and resuspended in 500 µL water. Samples were split evenly, and one set was treated with 5 mg/mL Cellulysin cellulase (Sigma) dissolved in water, while the other set was treated with water. After 30 minutes at 37°C, cells were pelleted and resuspended in 1 M oxalic acid. Total heme was measured as described above without the overnight treatment in 20 mM oxalic acid.

### *Mycobacterium abscessus* proteomics

#### Cell growth for proteomics

Mabs cells were grown in SCFM with or without 25 µM heme for 48 hours. Cells were collected by centrifugation and washed with water to remove media and unattached heme. Pellets were frozen at −80°C and stored for analysis. Three biological replicates each for the SCFM and SCFM +25 µM heme were measured.

#### Global quantitative proteomics of Mycobacterium abscessus

For global quantitative proteomics of Mabs samples, data-dependent acquisition-based proteomics was used. In brief, cells were lysed in urea lysis buffer (8 M urea, 100 mM Tris-HCl pH 8.5, and protease inhibitors [1 mini-Complete EDTA-free tablet]). The lysate was cleared by centrifugation at 14,000 rpm for 30 minutes at 4°C. The supernatant was transferred to a new tube, and the protein concentration was determined using a bicinchoninic acid (BCA) assay (Pierce). Subsequently, 10 µg of total protein was subjected to disulfide bond reduction with 10 mM dithiothreitol (at 56°C for 30 minutes) followed by alkylation with 10 mM iodoacetamide (at room temperature for 30 minutes in the dark). Excess iodoacetamide was quenched with 5 mM DTT (at room temperature for 15 minutes in the dark). Samples were then diluted sixfold with 50 mM ammonium bicarbonate and digested overnight at 37°C with a Trypsin/Lys-C mix (1:100). The next day, digestion was stopped by the addition of 1% trifluoroacetic acid (final [vol/vol]), followed by centrifugation at 14,000 × g for 10 minutes at room temperature to pellet precipitated lipids. Cleared digested peptides were desalted on an SDB-RPS Stage-Tip disk (49) and dried down in a speed-vac. Peptides were resuspended in 10 µL of 3% acetonitrile/0.1% formic acid and injected on Thermo Scientific Orbitrap Fusion Tribrid mass spectrometer with data independent acquisition method for peptide tandem mass spectrometry (MS/MS) analysis (50).

#### LC-MS/MS analysis

The UltiMate 3000 UHPLC system (Thermo Scientific) and EASY-Spray PepMap RSLC C18 25 cm × 75 µm ID column (Thermo Fisher Scientific) coupled with Orbitrap Fusion (Thermo) were used to separate fractioned peptides with a 5%–30% acetonitrile gradient in 0.1% formic acid over 70 min at a flow rate of 250 nL/min. After each gradient, the column was washed with 90% buffer B for 5 min and re-equilibrated with 98% buffer A (0.1% formic acid and 100% HPLC-grade water) for 30 min. Peptide MS/MS analysis was performed using a Thermo Scientific Orbitrap Fusion Tribrid mass spectrometer.

Survey scans of peptide precursors were conducted from 350 to 1,500 m/z at a resolution of 120,000 FWHM (at 200m/z), with an ion count target of 4 × 10^5^ and a maximum injection time of 50 ms. The instrument operated in top-speed mode with 3-second cycles for survey and MS/MS scans. Following each survey scan, tandem MS was carried out on the most abundant precursors, selected based on charge states from 2 to 6 and an intensity threshold greater than 5 × 10^4^. These precursors were isolated in the quadrupole with a 1.6 Th isolation window. Higher-energy collisional dissociation (HCD) fragmentation was applied with 30% collision energy, and the resulting fragments were detected using the rapid scan rate in the ion trap. The automatic gain control target for MS/MS was set to standard, and the maximum injection time was set to auto. Dynamic exclusion was enabled for 60 seconds with a 10-ppm mass tolerance around the precursor and its isotopes. Monoisotopic precursor selection was also enabled.

#### Data analysis

Raw mass spectrometric data were analyzed using the MaxQuant environment v.2.6.1.0 and employed Andromeda for database search at default settings with a few modifications (50, 51). The default was used for the first search tolerance and main search tolerance: 20 and 6 ppm, respectively. MaxQuant was set up to search with the reference *Mycobacterium abscessus* proteome database downloaded from Uniprot. MaxQuant performed the search trypsin digestion with up to two missed cleavages. Peptide, site, and protein false discovery rate were all set to 1% with a minimum of one peptide needed for identification, and LFQ was performed with a minimum ratio count of 1. The following modifications were used as variable modifications for identifications and included for protein quantification: oxidation of methionine (M), acetylation of the protein N-terminus, and deamination for asparagine or glutamine (NQ). Results obtained from MaxQuant were further analyzed using GraphPad Prism using multiple *t*-tests. Proteins identified as having a *P*-value < 0.05 and greater than equal to twofold change are listed in the tables. Fold change was calculated using normalized LFQ intensity values, and data used for analysis are included in File S1. The mass spectrometry proteomics data have been deposited to the ProteomeXchange Consortium via the PRIDE partner repository (52) with the data set identifier PXD057673.

#### Pyruvate assay

Cells were grown in SCFMGlu, and pyruvate secretion into the media was measured using the Pyruvate Assay Kit from Sigma (MAK332). Assay was performed as directed with the exception that 100 µL of media was used for pyruvate measurements. Water and SCFMGlu background gave similar signals, so water was used for background measurements. Fluorescence was measured on a Tecan Spark plate reader with excitation at 530 nm and emission at 585 nm.

### *Mycobacterium abscessus* untargeted metabolomics

Untargeted metabolomics was performed by Creative Proteomics (Shirley, NY) which is a commercial facility. Methods provided by Creative proteomics are as previously described (35, 36). Briefly, metabolites were extracted with 300 µL of 80% methanol. All samples were vortexed with sufficient mixing and followed by sonication for 30 min, 4°C. Each sample was kept at −20°C for 1 h, then vortexed for 30 s, and kept at 4°C for 15 min. After that, samples were centrifuged at 12,000 rpm and 4°C for 10 min. Finally, 200 µL of supernatant and 5 µL of DL-o-chlorophenylalanine (0.14 mg/mL) were transferred to the vial for LC-MS analysis. Quality control (QC) samples were used to evaluate the methodology. The same amount of extract was obtained from each sample and mixed as QC samples.

Separation was performed by a Vanquish Flex UPLC combined with Q Exactive plus MS (Thermo) and was screened with Electrospray ionization (ESI)-MS. The LC system comprises an ACQUITY UPLC HSS T3 (100 × 2.1 mm × 1.8 µm) with UPLC. Mass scan mode: full scan (m/z 70–1,050 and resolution: 70,000) and dd-MS2 (TopN = 10 and resolution: 17,500); collision mode: HCD; mass spectrometry parameters in ESI+ and ESI− mode are listed as follows:

ESI+: heater temperature 300°C; sheath gas flow rate, 45 arb; Aux gas flow rate, 15 arb; sweep gas flow rate, 1 arb; spray voltage, 3.0 kV; capillary temperature, 350°C; S-Lens RF Level, 30%.

ESI−: heater temperature 300°C; sheath gas flow rate, 45 arb; Aux gas flow rate, 15 arb; sweep gas flow rate, 1 arb; spray voltage, 3.2 kV; capillary temperature, 350°C; S-Lens RF Level, 60%.

All peaks in ESI+ were merged and imported into the SIMCA-P software for multivariate statistical analysis. To investigate the global metabolism variations, PCA was used to analyze all observations acquired in both ion modes. The partial least-squares discriminant analysis (PLS-DA) score plot or orthogonal partial least squares discriminant analysis (OPLS-DA) score plot both show ed a clear separation of the treated and untreated groups (Fig. S12).

Metabolites identified in positive and negative mode were analyzed for quantitative enrichment using Metaboanalyst (https://www.metaboanalyst.ca/). Metabolic pathways from KEGG (https://www.kegg.jp/kegg/pathway.html) for *Mycobacterium tuberculosis* H37Rv were used to identify other metabolites in the pathway and for metabolite pathway maps in figures.

Data used in analysis are included in Files S2 and S3.

### Microscopy of *Mycobacterium abscessus* biofilms

Mabs biofilms were grown in 1 mL media as indicated in MatTek CCS-4 four-well Chambered Cell Culture Slides for 1 week at 37°C. Media was removed, and films were washed with PBS. Films were stained with 250 µL AO (Thermo Scientific) for 10 minutes, at RT in the dark, washed with PBS 3×, followed by 30 minutes with 1 mg/mL CW (Sigma) and again washed 3× with PBS. Films were blocked with 1% BSA in PBS for 1 hour at RT followed by anti-HuHb (Invitrogen HB11-201.11) at 1:1,000 in 0.1% BSA overnight at 4°C. Films were washed 3× in PBS, and the secondary antibody (Goat anti-Mouse IgG1 Cross-Adsorbed Secondary Antibody, Alexa Fluor 647, Invitrogen) was added at 1:2,000 in 0.1% BSA in PBS for 1 hour at RT. Films were washed 3× with PBS, chambers were removed, and films were allowed to dry 1–2 hours at RT before glass slide was affixed. Films were visualized using a Nikon Ti2 inverted microscope with a 60× oil objective. The DAPI (4′,6-diamidino-2-phenylindole) filter was used for CW (CW ex. 380 nm and em. 475 nm), fluorescein isothiocyanate (FITC) filter for AO (AO ex. 502 and em. 525), and the Cyt5 filter for anti-Hb (ex. 650 nm and em. 665 nm). Both Hb treated and untreated were probed and imaged for anti-Hb, but only treated wells had fluorescence above background in the Cyt5 channel (data not shown). A *z*-stack of 1 µM slices is averaged for the images. For quantification, background was measured in untreated films and subtracted from the AO and CW channels. The microscope was purchased with funds from National Science Foundation grant DBI-1828264.

## SUPPLEMENTAL MATERIAL

Data S1 spectrum.02415-24-s0001.xlsx Proteomics data used for Tables 1, S1, and S2.

Data S2 spectrum.02415-24-s0002.xlsx Metabolomics data used for analysis-negative mode.

Data S3 spectrum.02415-24-s0003.xlsx Metabolomics data used for analysis-positive mode.

Supplemental material spectrum.02415-24-s0004.docx Tables S1 and S2; Fig. S1 to S13.

ASM does not own the copyrights to Supplemental Material that may be linked to, or accessed through, an article. The authors have granted ASM a non-exclusive, world-wide license to publish the Supplemental Material files. Please contact the corresponding author directly for reuse.
