# Supplementary material for: Heme alters biofilm formation in Mycobacterium abscessus
Source: Microbiol Spectr. 2024 Dec 23;13(2):e02415-24. doi: 10.1128/spectrum.02415-24 (PMC11792503; doi:10.1128/spectrum.02415-24)
Supplement: Supplemental material — Tables S1 and S2; Fig. S1 to S13. [file spectrum.02415-24-s0004.docx]

**Supplemental Tables**

| Supplemental Table 1. *Mycobacterium abscessus* proteins significantly decreased in heme treatment in SCFM compared to untreated | | | |
| --- | --- | --- | --- |
| Protein function | **Gene ID** | **log2 fold change** | **P value*** |
| Mycobactin synthesis (MbtF) | MAB_2123 | -5.3 | 0.0124 |
| ESX-3 (eccB3) | MAB_2233c | -4.7 | 0.0018 |
| ESX-3 | MAB_2234c | -4.4 | 0.0001 |
| ESX-3 | MAB_2226c | -3.2 | 0.0137 |
| ESX-3 | MAB_2232c | -2.9 | 0.0011 |
| ESX-3 | MAB_2227c | -2.5 | 0.0104 |
| Mycobactin synthesis (MbtB ) | MAB_2121c | -2.4 | 0.0067 |
| Mycobactin uptake (IrtB) | MAB_2261c | -1.7 | 0.0001 |
| ESX-3 | MAB_2229c | -1.4 | 0.0197 |
| Putative transcriptional regulator, PadR-like | MAB_0383c | -1.6 | 0.0463 |
| Putative PE12 homolog | MAB_2679 | -2.1 | 0.0139 |
| Hypothetical Protein | MAB_1013 | -1.8 | 0.0161 |
| Proteins with measurable expression in SCFM but not SCFM + Heme** | | | |
| Mycobactin uptake (IrtA) | **MAB_2262c** | **NA** | **NA** |
| ESX-3 | **MAB_2225c** | **NA** | **NA** |
| Mycobactin synthesis (MbtC) | **MAB_2120c** | **NA** | **NA** |
| Mycobactin synthesis (MbtI) | **MAB_2245** | **N/A** | **N/A** |
| Probable sulfite reductase | MAB_2492 | N/A | N/A |
| Hypothetical protein | MAB_0367c | N/A | N/A |
| Hypothetical protein | MAB_4000 | N/A | N/A |
| Putative enoyl-CoA hydratase/isomerase | MAB_0834 | N/A | N/A |
| Glycosyltransferase | MAB_4694c | N/A | N/A |
| *P-value was calculated using multiple t-tests 21 total proteins were identified that had ≥ 2-fold change in expression and a p-value <0.05. **An additional 9 proteins were measured in all 3 SCFM samples but were below detection in all 3 heme-treated samples. Proteins annotated as involved in iron homeostasis are in bold and also listed Table 1 in the text. | | | |

| Supplemental Table 2. *Mycobacterium abscessus* proteins significantly increased in heme treatment in SCFM compared to untreated | | | |
| --- | --- | --- | --- |
| Protein function | **Gene ID** | **log_2_ (fold change)** | **P-value*** |
| Hypothetical protein | MAB_2345 | 2.2 | 0.0414 |
| Hypothetical protein | MAB_0551 | 1.6 | 0.0391 |
| MmpL family protein | MAB_1287 | 1.5 | 0.0332 |
| Putative transporter | MAB_2780c | 1.5 | 0.0493 |
| AraC transcriptional regulator | MAB_4712 | 1.3 | 0.0497 |
| UDP-glucose epimerase GalE1 | MAB_4003c | 1.2 | 0.0090 |
| Hypothetical protein | MAB_2220c | 1.2 | 0.0410 |
| Mn transport protein MntH | MAB_1031c | 1.1 | 0.0103 |
| DUF1942 domain protein | MAB_0462c | 1.1 | 0.0154 |
| Proteins with measurable expression in SCFM + Heme but not SCFM ** | | | |
| Hypothetical protein | MAB_0325c | N/A | N/A |
| Conserved hypothetical protein | MAB_3609 | N/A | N/A |
| Putative nicotinamidase | MAB_1472c | N/A | N/A |
| Putative dioxygenase | MAB_0303 | N/A | N/A |
| Hypothetical protein | MAB_3464 | N/A | N/A |
| Hypothetical protein | MAB_1936 | N/A | N/A |
| Putative oxidoreductase | MAB_1127c | N/A | N/A |
| Probable lipoprotein aminopeptidase LpqL | MAB_4212c | N/A | N/A |
| Hypothetical protein | MAB_0112 | N/A | N/A |
| *P-value was calculated using multiple t-tests, proteins were identified that had ≥ 2-fold change in expression and a p-value <0.05. ** An additional 9 proteins were measured in all 3 heme-treated samples but were below detection in all 3 SCFM samples. | | | |

**Supplemental Figures**


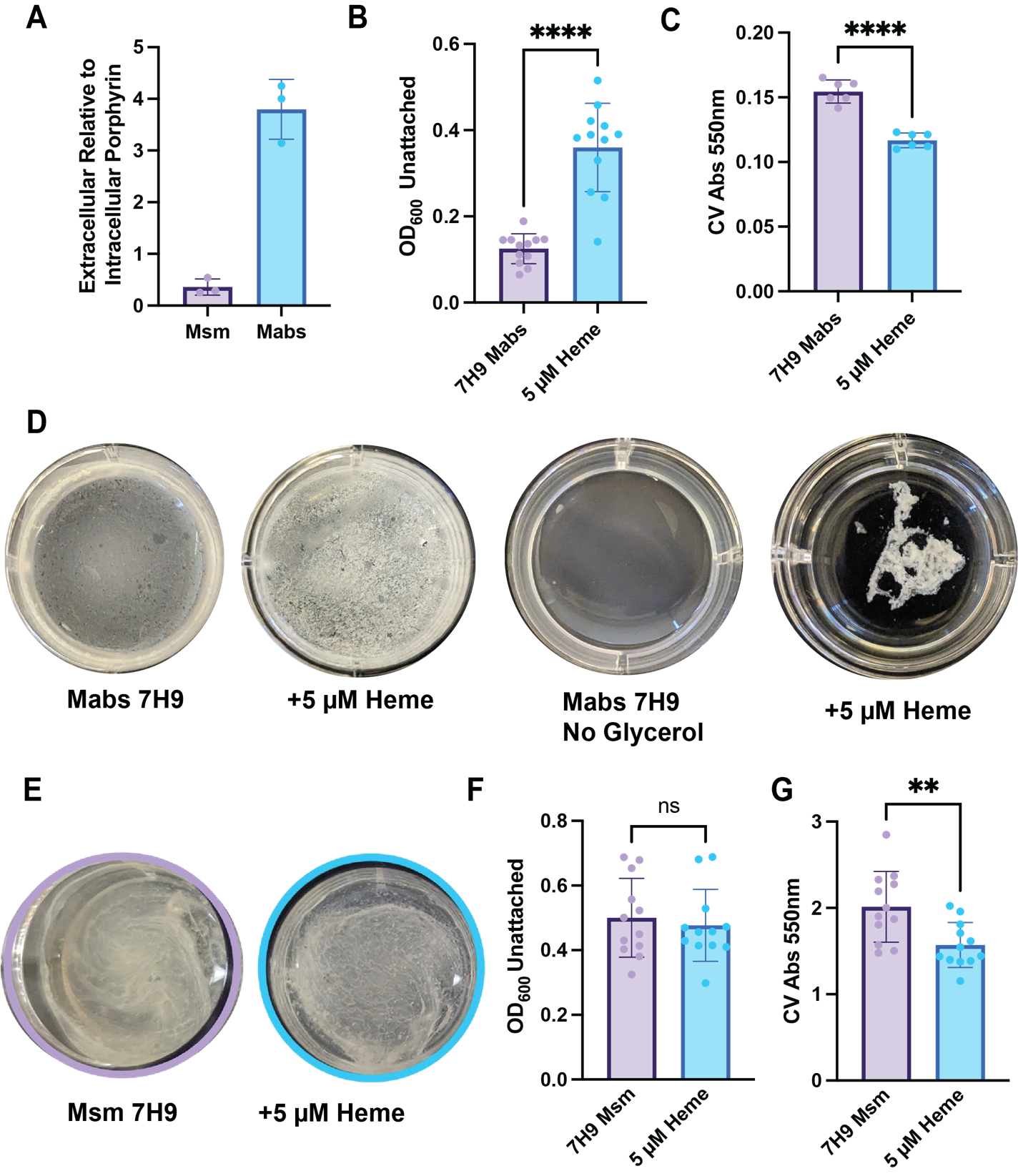


**Figure S1. The effect of heme on Msm biofilm and Mabs submerged film. A.** Ratio of extracellular porphyrin AFU (654nm) to intracellular porphyrin AFU in Mab and Msm. **B.** Unattached Mabs cells grown in 96-well plates in 7H9 media treated with heme or Hb measured by optical density. **C**. Attached biofilm measured via crystal violet (CV) assay in same 96-well plate as in (**B**). **D.** Representative images of Mabs pellicles grown in 7H9 (No BSA or Tween) with and without glycerol added and treated with 5 µM heme. **E.** Representative images of Msm pellicles grown in 7H9 (No BSA or Tween) treated with 5 µM heme. **F.** Unattached Msm cells grown in 96-well plates in 7H9 media treated with 5 µM heme measured by optical density. **G**. Attached biofilm measured via crystal violet (CV) assay in same 96-well plate as in (**F**). Statistical analysis **in B, C, F** and **G** was measured via a two-tailed paired Student’s *t* test was used and the calculated ****P values < 0.0001, ns = 0.6261 and **P = 0.0046.


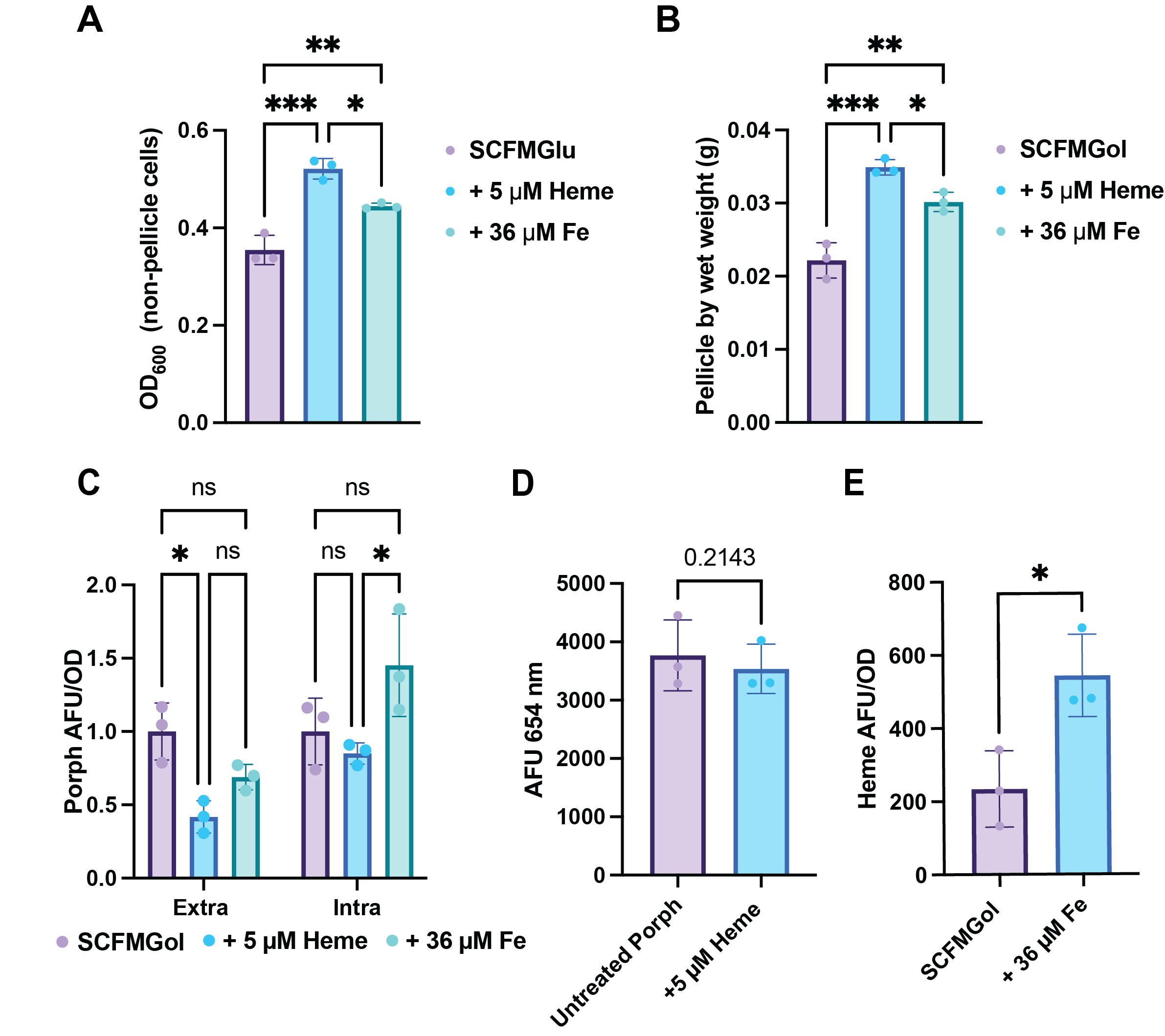


**Figure S2. The effects of heme and iron on Mabs pellicle formation and metabolite secretion. A.** Cell growth measure by OD_600_ of Mabs cells grown in SCFMGlu. Cells were grown in SCFMGlu and treated with 5 µM heme or 36 µM iron (Fe)**.** Cells measured were either unattached aggregates or submerged film. **B.** Pellicle formation by Mabs in SCFMGol measured by wet weight. Cells were grown in SCFMGol and treated with 5 µM heme or 36 µM iron (Fe). **C.** Secreted porphyrin (extra) and intracellular porphyrin (intra) measured for Mabs cells grown in SCFMGol treated with 5 µM heme or 36 µM iron (Fe) in 12 well plates. Porphyrin fluorescence is normalized to optical density and relative to untreated SCFMGol for comparison. **D.** Measurement of inner filter effect of 5 µM heme on porphyrin fluorescence. Porphyrin fluorescence of media from Mabs cells grown in SCFMGol was measured (Untreated Porph) then 5 µM heme was added and porphyrin fluorescence of media was measured again (+ 5 µM Heme). **E.** Total heme fluorescence of Mabs cells grown in SCFMGol (3.6 µM Fe added in formulation) and with 36 µM iron added (+36 µM Fe). Statistical analysis in **A** and **B** was assessed by one-way analysis of variance (ANOVA) with Tukey's multiple comparisons test. For **A**, Calculated P values were, ***P = 0.0002, **P= 0.0051, and *P = 0.0110. For **B**, Calculated P values were, ***P = 0.0002, **P= 0.0028, and *P = 0.0327. Statistical analysis in **C** was assessed by two-way analysis of variance (ANOVA) with a Sidak’s multiple comparisons test. Calculated P values were, *P = 0.0222 and *P= 0.0176, respectively. Statistical significance in **D** and **E** was measured by a two-tailed paired Student’s *t* test, P= 0.2143 and *P=0.0253, respectively.

**
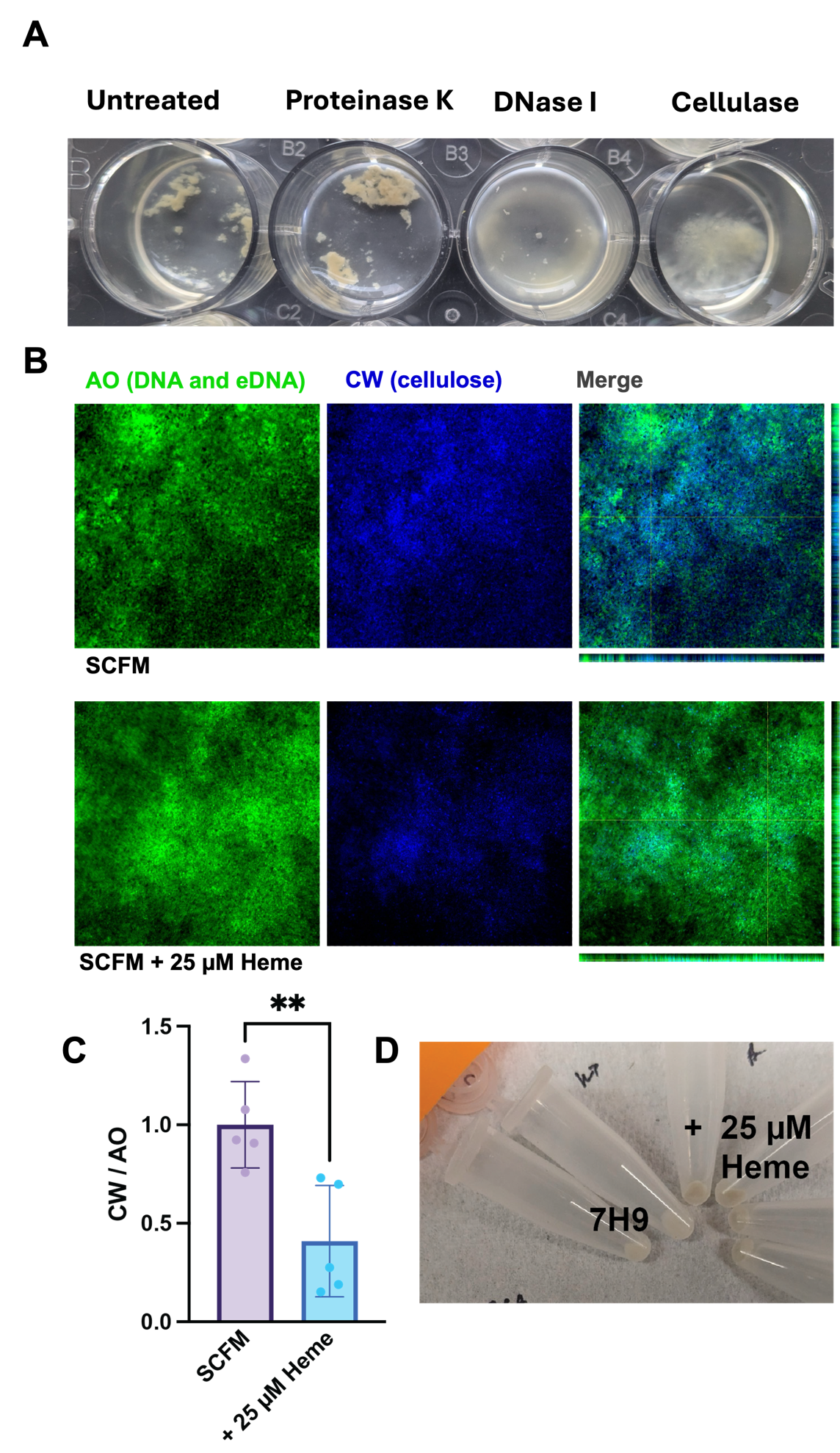
**

**Figure S3. Cellulose and eDNA are structural components in biofilms of Mycobacterium abscessus A.** Image of Mabs cell aggregates treated with water (untreated), Proteinase K, DNase I or Cellulase. **B.** Representative confocal microscopy images of Mabs biofilms in SCFM (top) and SCFM + 25 µM Heme (bottom). Films were stained with acridine orange (AO) for eDNA and DNA which stains total biofilm and calcofluor white (CW) for cellulose. The merged panel includes orthogonal views along the yellow lines in the panel. **C.** Quantification of AO and CW intensity in SCFM and SCFM + 25 µM Heme **D.** Image of washed Mabs cell pellets grown in 7H9 and 7H9 + 25 µM heme. Statistical significance in **C** was measured by a two-tailed unpaired Student’s *t* test, P value calculated was **P= 0.0061.


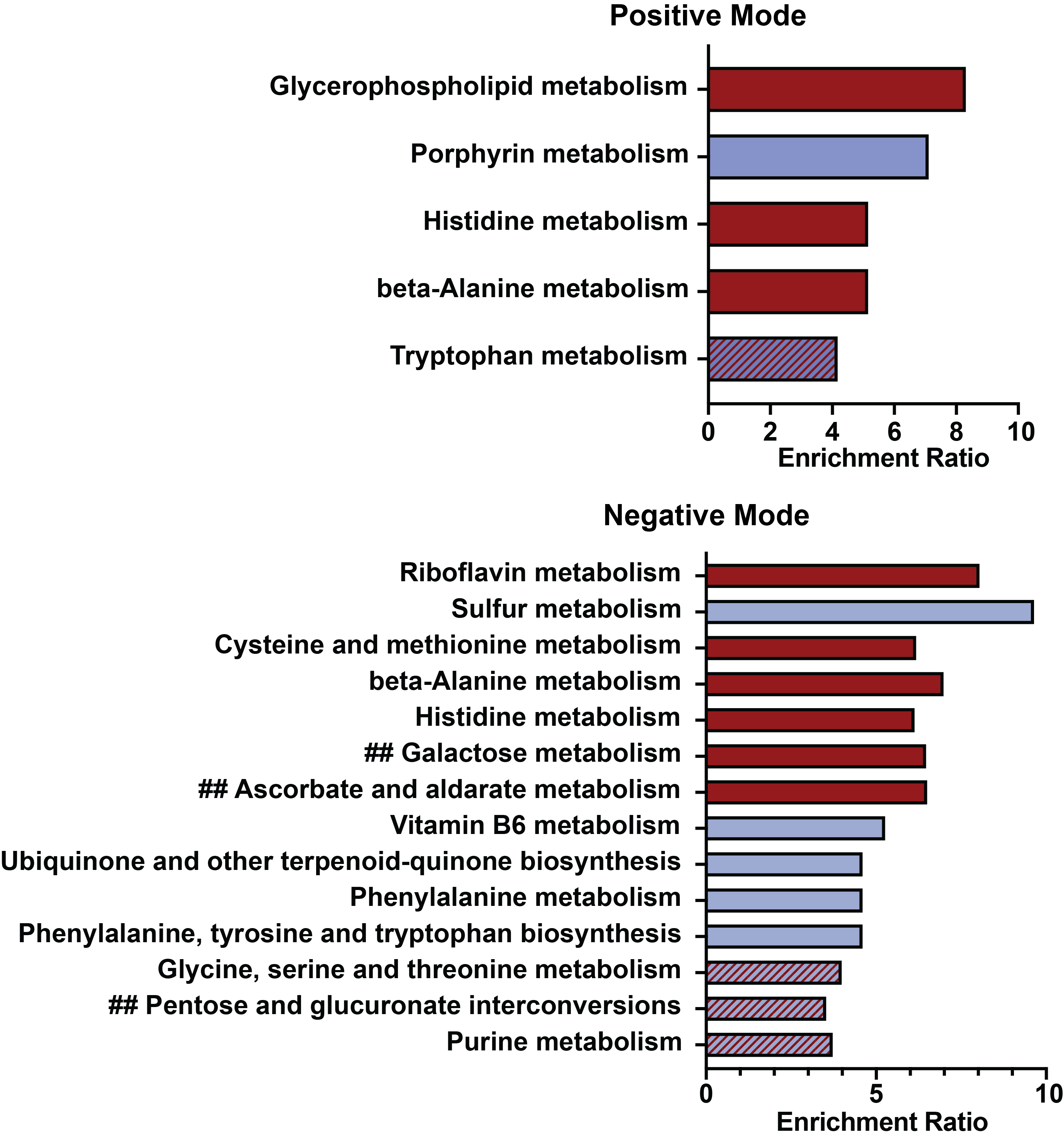


**Figure S4 enrichment of measured and identified metabolites calculated by Metaboanalyst using KEGG database selection. A.** Metabolites identified in positive mode. Red coloring signifies metabolites enriched in heme treatment, blue coloring signifies metabolites enriched in SCFM relative to heme and stripes indicate enrichment of some metabolites in both heme and SCFM. P value calculated for Tryptophan metabolism enrichment via Metaboanalyst was = 0.023812, p values of all other sets shown is < 0.023812. **B.** Metabolites identified in negative mode. Red coloring signifies metabolites enriched in heme treatment, blue coloring signifies metabolites enriched in SCFM relative to heme and stripes indicate enrichment of some metabolites in both heme and SCFM. ** P value calculated for Purine metabolism enrichment via Metaboanalyst was =0.029289, p values of other sets are all < 0.029289. ## Indicates pathways with UDP-Glucose as an enriched metabolite.


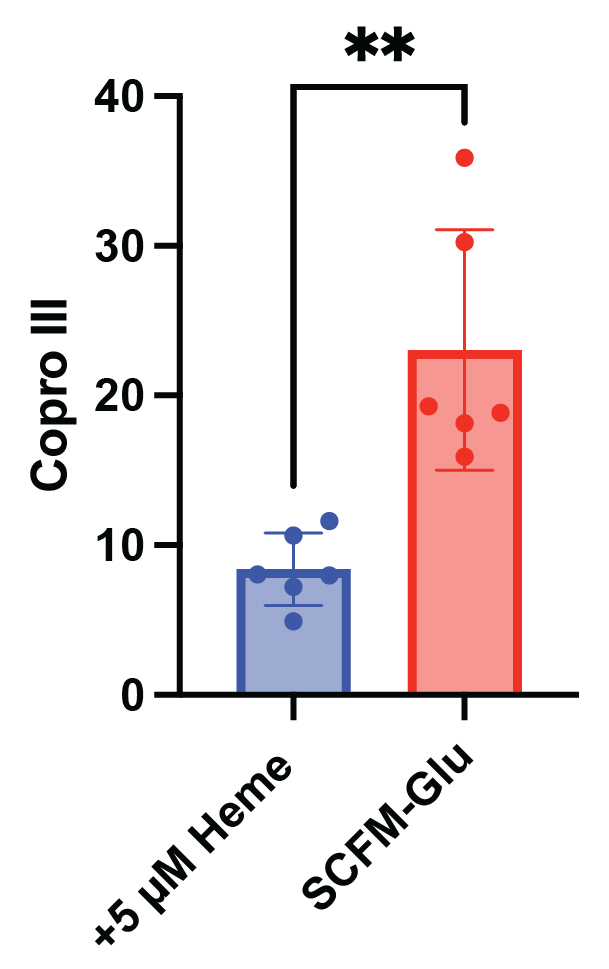


**Figure S5. Coproporphyrin levels in Mabs measured by metabolomics.** Cells were grown in SCFMGlu +/- 5 µM heme. Statistical significance was measured by a two-tailed unpaired Student’s *t* test, P value calculated was **P = 0.0016.


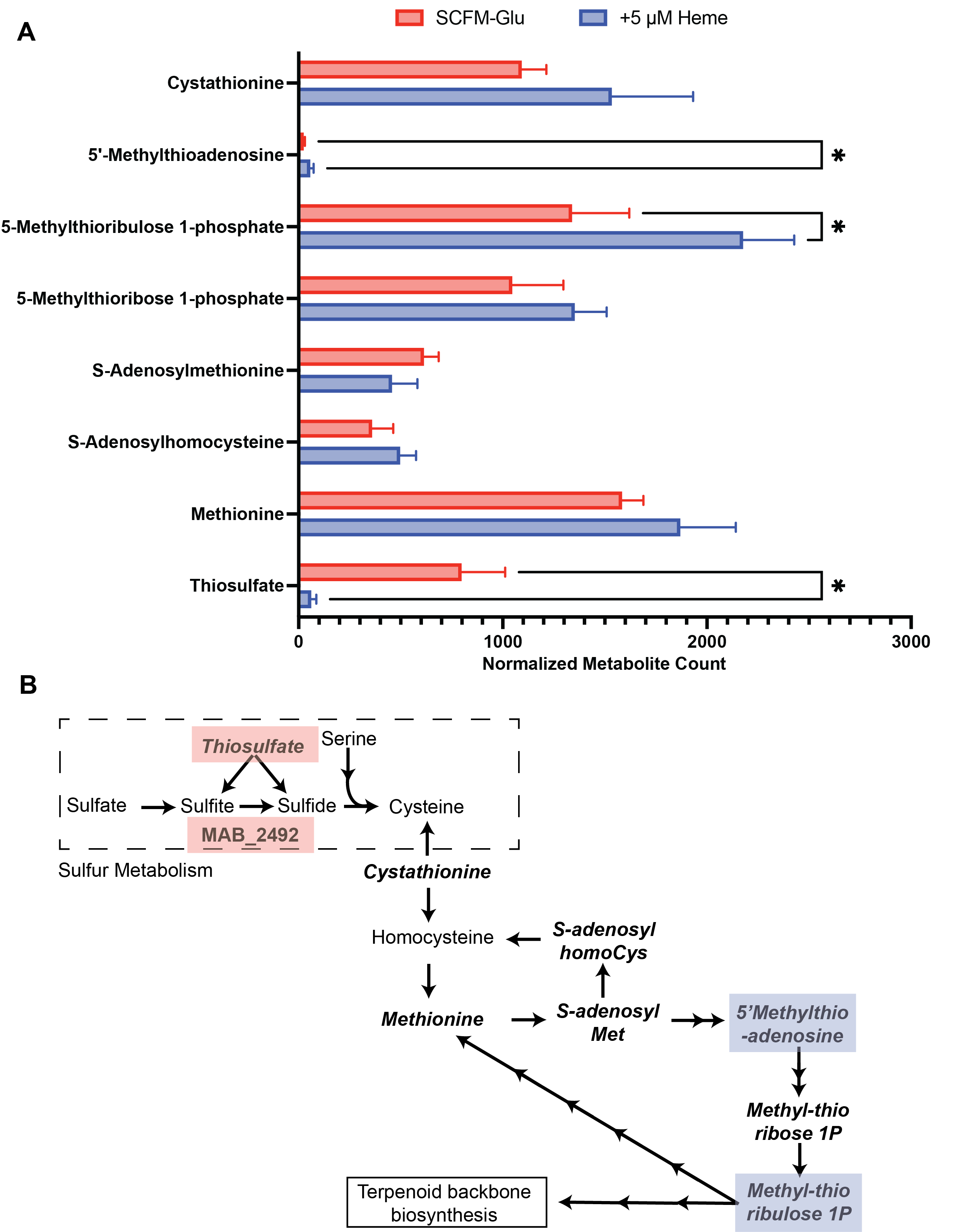


**Figure S6**. **Metabolite levels of sulfur and methionine metabolism pathways** **A**. Levels of metabolites in Mabs cells grown in SCFMGlu (red) and SCFMGlu + 5 µM heme (blue). **B**. Simplified pathway with metabolites enriched in heme treatment colored by blue box and metabolites and pathway enzymes reduced in heme treatment colored by red box. Other measured metabolites in pathway are bolded. P-values were calculated by multiple t-test. *P values are < 0.05.


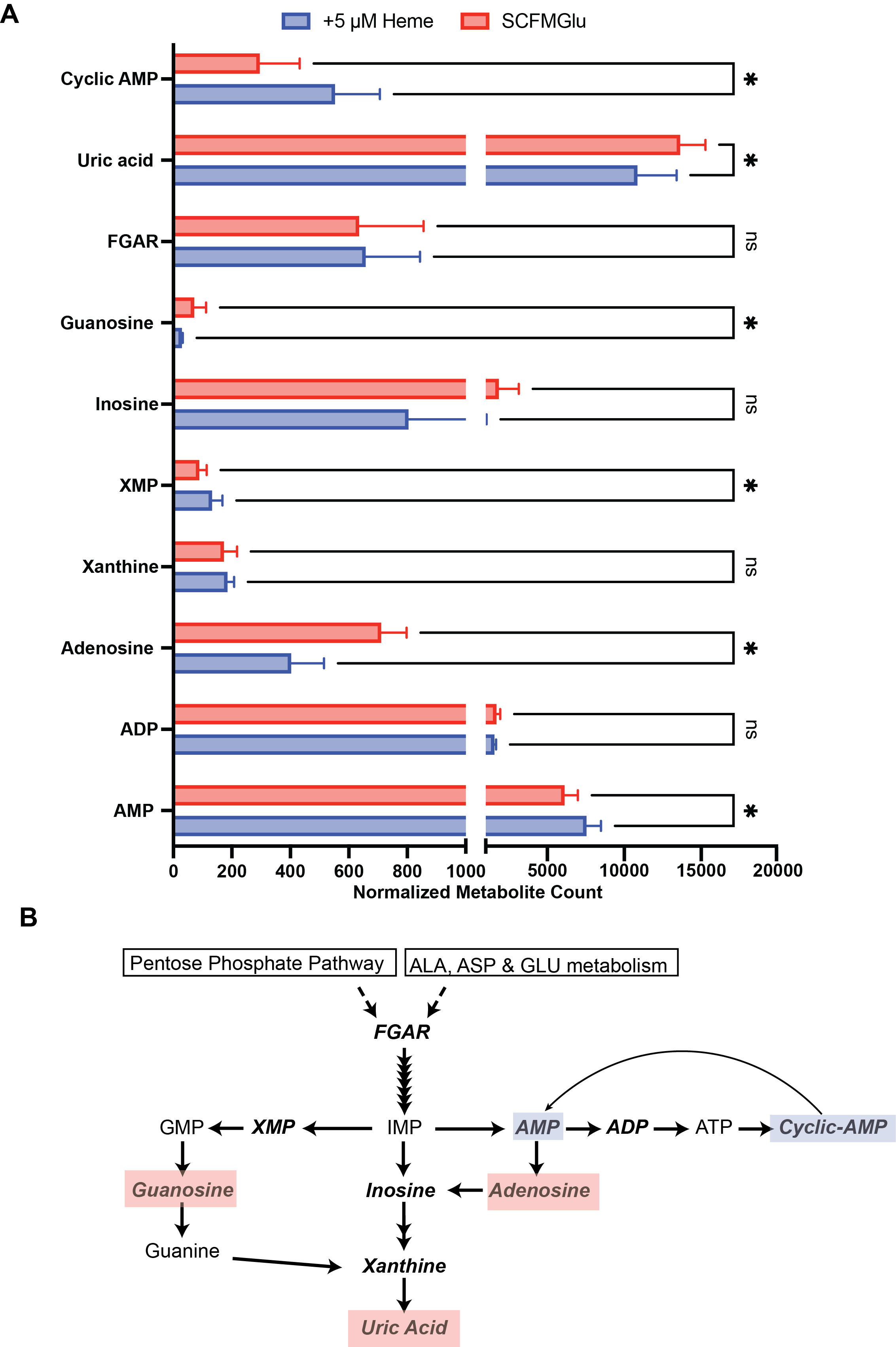


**Figure S7**. **Metabolite levels of the purine metabolism pathway** **A**. Levels of metabolites in Mabs cells grown in SCFMGlu (red) and SCFMGlu + 5 µM Heme (blue). **B**. Simplified pathway with metabolites enriched in heme treatment colored by blue box and metabolites and pathway enzymes reduced in heme treatment colored by red box. Other measured metabolites in pathway are bolded. P-values were calculated by multiple t-test. *P values are < 0.05.


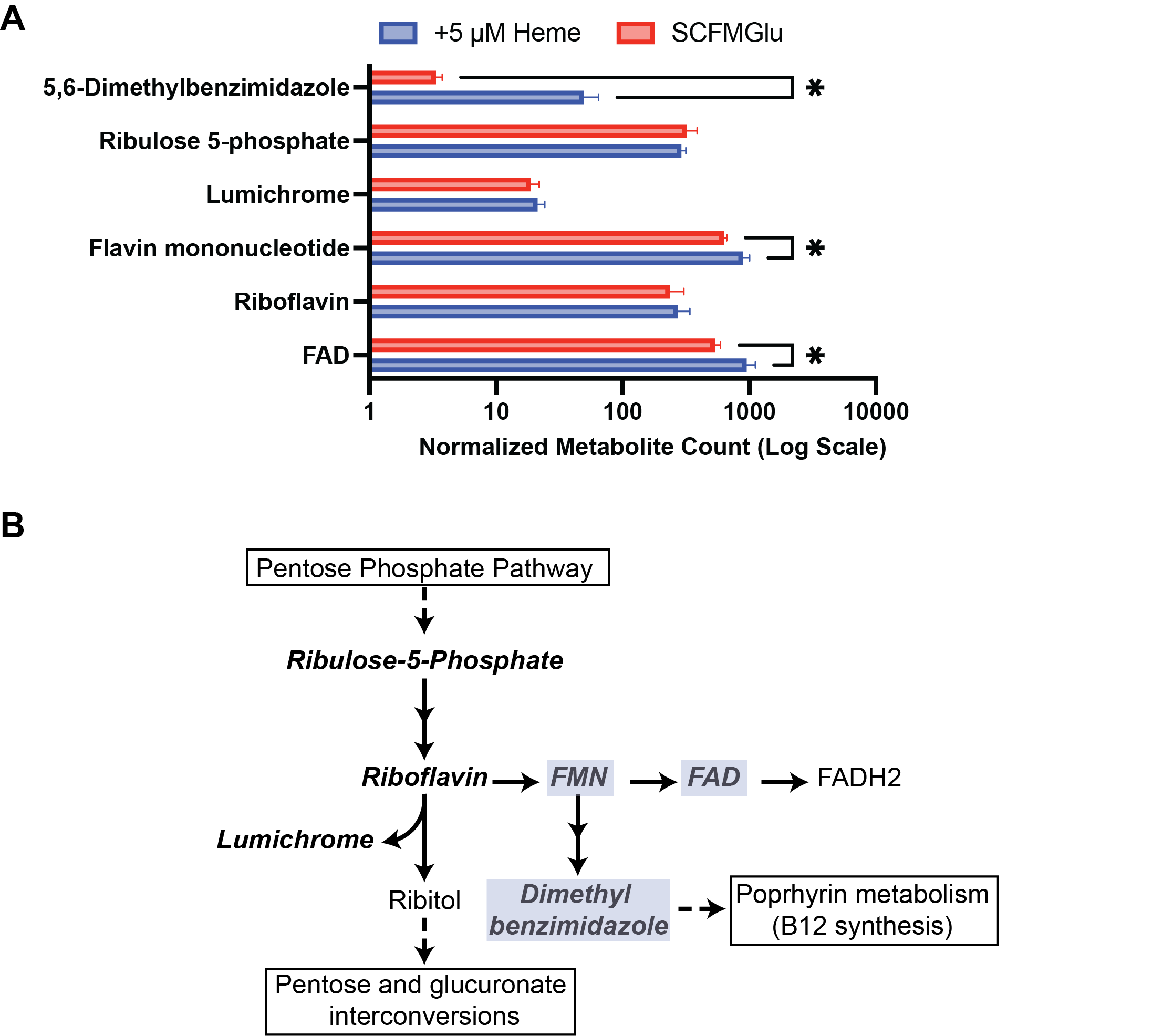


**Figure S8**. **Metabolite levels of the riboflavin metabolism pathway** **A.** Levels of metabolites in Mabs cells grown in SCFMGlu (red) and SCFMGlu+ 5 µM Heme (blue). **B.** Simplified pathway with metabolites enriched in heme treatment colored by blue box and metabolites and pathway enzymes reduced in heme treatment colored by red box. Other measured metabolites in pathway are bolded. P-values were calculated by multiple t-test. *P values are < 0.05.


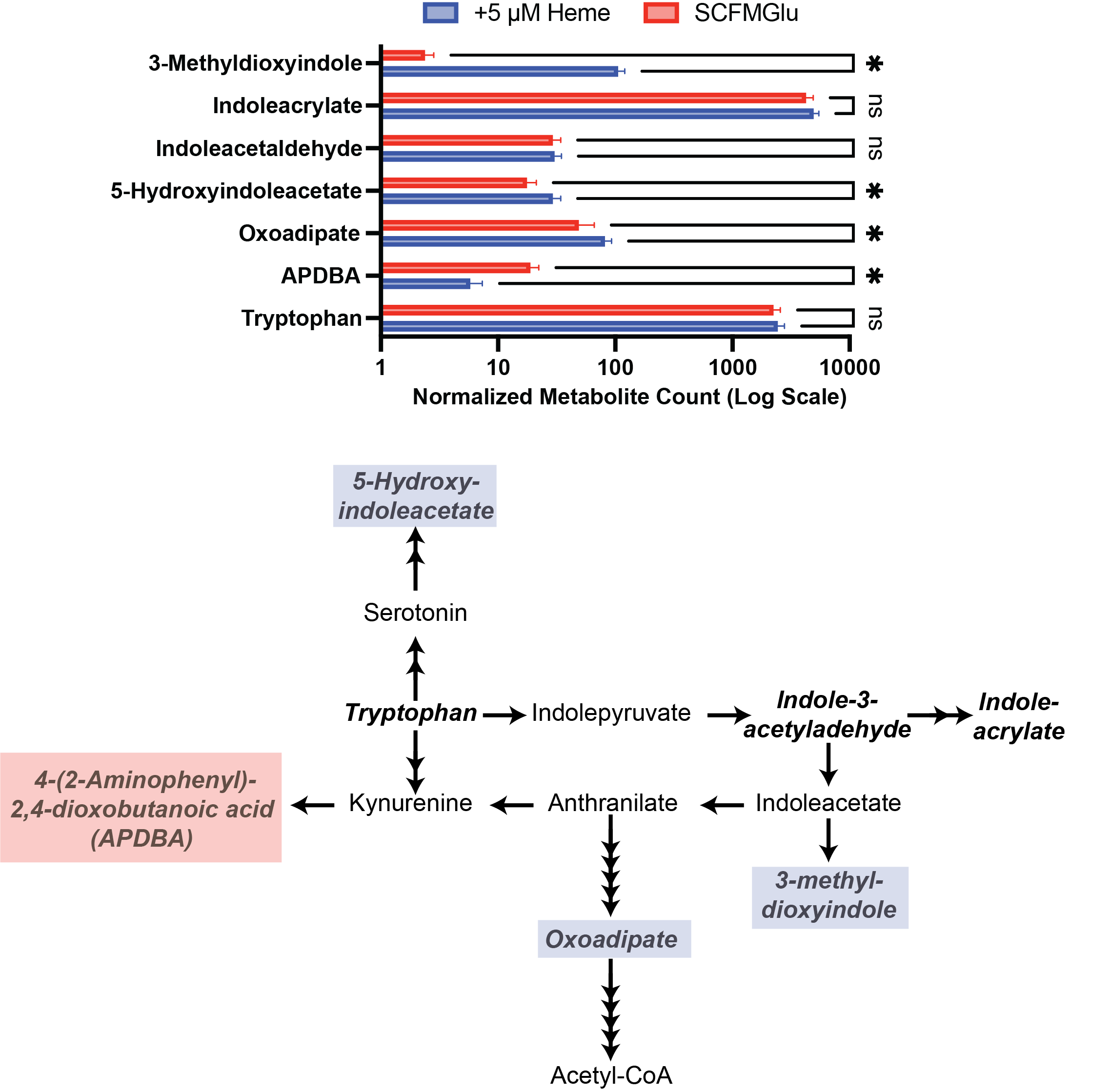


**Figure S9**. **Metabolite levels of the tryptophan metabolism pathway.** **A.** Levels of metabolites in Mabs cells grown in SCFMGlu (red) and SCFMGlu+ 5 µM Heme (blue). **B.** Simplified pathway with metabolites enriched in heme treatment colored by blue box and metabolites and pathway enzymes reduced in heme treatment colored by red box. Other measured metabolites in pathway are bolded. P-values were calculated by multiple t-test. *P values are < 0.05.


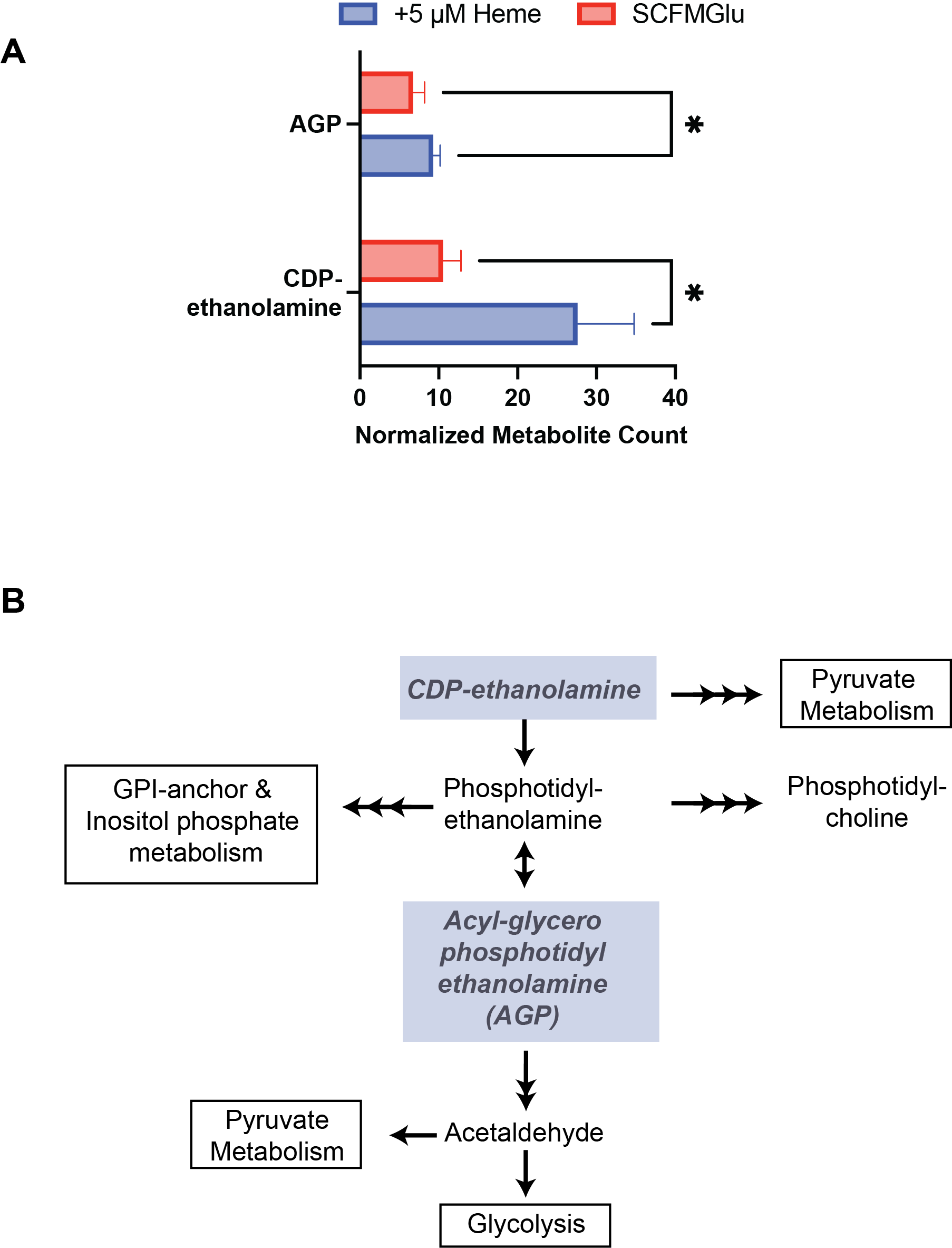


**Figure S10**. **Metabolite levels of the glycerophospholipid metabolism pathway. A.**  Levels of metabolites in Mabs cells grown in SCFMGlu (red) and SCFMGlu+ 5 µM Heme (blue). **B.** Simplified pathway with metabolites enriched in heme treatment colored by blue box. P-values were calculated by multiple t-test. *P values are < 0.05.


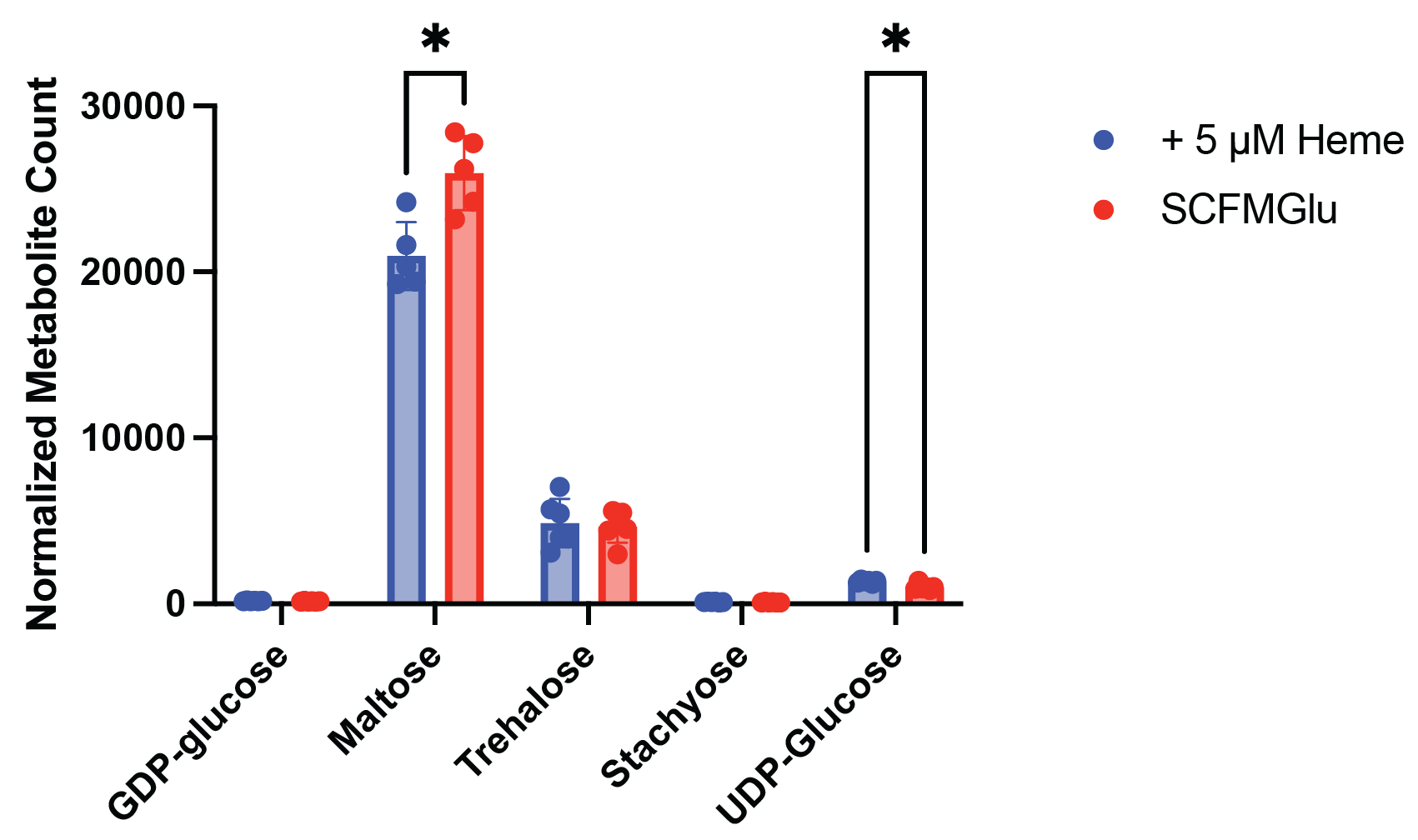


**Figure S11. Metabolite levels UDP-Glucose and other sugars.** Levels of metabolites in Mabs cells grown in SCFMGlu (red) and SCFMGlu+ 5 µM Heme (blue). P-values were calculated by multiple t-test. *P values are < 0.05.


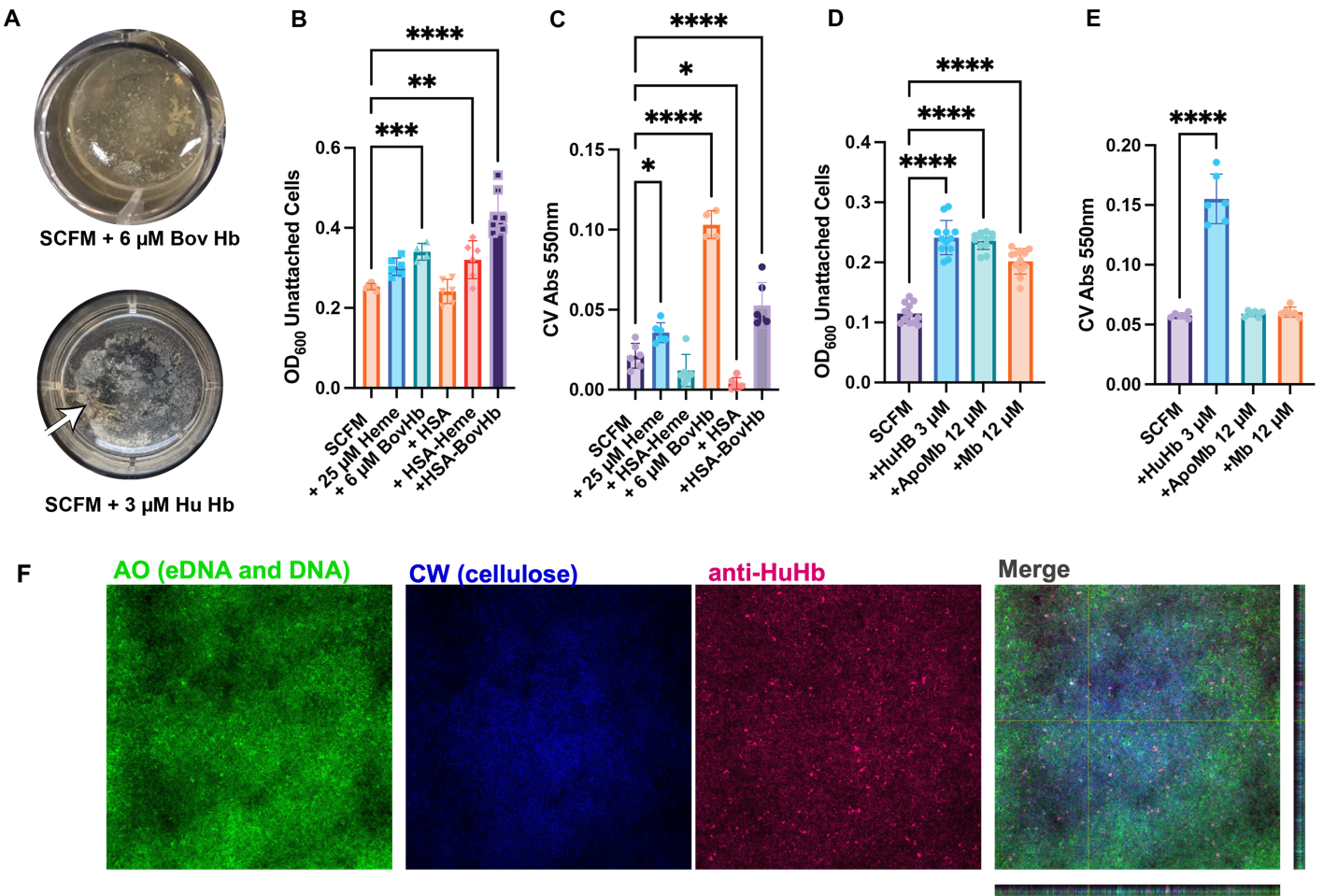


**Figure S12. The effect of Hb on Mabs biofilm formation. A**. Representative images of Mabs biofilm formation in SCFM with 6 µM BovHb and 3 µM Hu Hb. Arrow in HuHb well indicates HuHb aggregates **B**. The effect of heme, BovHb and human serum albumin (HSA) on Mabs unattached cell growth measured by optical density and (**C**) attached biofilm formation measured by crystal violet (CV) assay in SCFM in 96 well plates. **D**. The effect of HuHb, apo-myoglobin (Apo-Mb) and myoglobin (Mb) on Mabs unattached cell growth measured by optical density and (**E**) attached biofilm formation measured by crystal violet (CV) assay in SCFM in 96 well plates. **F.** Mabs biofilm grown for 7 days in SCFM + 3 µM HuHb. Film was stained with DNA binding dye Acridine orange (AO) to approximate total biofilm as dye binds both intracellular and extracellular DNA and cellulose binding dye calcofluor white (CW). HuHb was detected using a monoclonal antibody for human hemoglobin (Invitrogen). The merged panel includes orthogonal views along the yellow lines in the panel.

**Supplemental Figure Captions**

**Figure S1. The effect of heme on Msm biofilm and Mabs submerged film. A.** Ratio of extracellular porphyrin AFU (654nm) to intracellular porphyrin AFU in Mab and Msm. **B.** Unattached Mabs cells grown in 96-well plates in 7H9 media treated with heme or Hb measured by optical density. **C**. Attached biofilm measured via crystal violet (CV) assay in same 96-well plate as in (**B**). **D.** Representative images of Mabs pellicles grown in 7H9 (No BSA or Tween) with and without glycerol added and treated with 5 µM heme. **E.** Representative images of Msm pellicles grown in 7H9 (No BSA or Tween) treated with 5 µM heme. **F.** Unattached Msm cells grown in 96-well plates in 7H9 media treated with 5 µM heme measured by optical density. **G**. Attached biofilm measured via crystal violet (CV) assay in same 96-well plate as in (**F**). Statistical analysis **in B, C, F** and **G** was measured via a two-tailed paired Student’s *t* test was used and the calculated ****P values < 0.0001, ns = 0.6261 and **P = 0.0046.

**Figure S2. The effects of heme and iron on Mabs pellicle formation and metabolite secretion. A.** Cell growth measure by OD_600_ of Mabs cells grown in SCFMGlu. Cells were grown in SCFMGlu and treated with 5 µM heme or 36 µM iron (Fe)**.** Cells measured were either unattached aggregates or submerged film. **B.** Pellicle formation by Mabs in SCFMGol measured by wet weight. Cells were grown in SCFMGol and treated with 5 µM heme or 36 µM iron (Fe). **C.** Secreted porphyrin (extra) and intracellular porphyrin (intra) measured for Mabs cells grown in SCFMGol treated with 5 µM heme or 36 µM iron (Fe) in 12 well plates. Porphyrin fluorescence is normalized to optical density and relative to untreated SCFMGol for comparison. **D.** Measurement of inner filter effect of 5 µM heme on porphyrin fluorescence. Porphyrin fluorescence of media from Mabs cells grown in SCFMGol was measured (Untreated Porph) then 5 µM heme was added and porphyrin fluorescence of media was measured again (+ 5 µM Heme). **E.** Total heme fluorescence of Mabs cells grown in SCFMGol (3.6 µM Fe added in formulation) and with 36 µM iron added (+36 µM Fe). Statistical analysis in **A** and **B** was assessed by one-way analysis of variance (ANOVA) with Tukey's multiple comparisons test. For **A**, Calculated P values were, ***P = 0.0002, **P= 0.0051, and *P = 0.0110. For **B**, Calculated P values were, ***P = 0.0002, **P= 0.0028, and *P = 0.0327. Statistical analysis in **C** was assessed by two-way analysis of variance (ANOVA) with a Sidak’s multiple comparisons test. Calculated P values were, *P = 0.0222 and *P= 0.0176, respectively. Statistical significance in **D** and **E** was measured by a two-tailed paired Student’s *t* test, P= 0.2143 and *P=0.0253, respectively.

**Figure S3. Cellulose and eDNA are structural components in biofilms of Mycobacterium abscessus A.** Image of Mabs cell aggregates treated with water (untreated), Proteinase K, DNase I or Cellulase. **B.** Representative confocal microscopy images of Mabs biofilms in SCFM (top) and SCFM + 25 µM Heme (bottom). Films were stained with acridine orange (AO) for eDNA and DNA which stains total biofilm and calcofluor white (CW) for cellulose. The merged panel includes orthogonal views along the yellow lines in the panel. **C.** Quantification of AO and CW intensity in SCFM and SCFM + 25 µM Heme **D.** Image of washed Mabs cell pellets grown in 7H9 and 7H9 + 25 µM heme. Statistical significance in **C** was measured by a two-tailed unpaired Student’s *t* test, P value calculated was **P= 0.0061.

**Figure S4 enrichment of measured and identified metabolites calculated by Metaboanalyst using KEGG database selection. A.** Metabolites identified in positive mode. Red coloring signifies metabolites enriched in heme treatment, blue coloring signifies metabolites enriched in SCFM relative to heme and stripes indicate enrichment of some metabolites in both heme and SCFM. P value calculated for Tryptophan metabolism enrichment via Metaboanalyst was = 0.023812, p values of all other sets shown is < 0.023812. **B.** Metabolites identified in negative mode. Red coloring signifies metabolites enriched in heme treatment, blue coloring signifies metabolites enriched in SCFM relative to heme and stripes indicate enrichment of some metabolites in both heme and SCFM. ** P value calculated for Purine metabolism enrichment via Metaboanalyst was =0.029289, p values of other sets are all < 0.029289. ## Indicates pathways with UDP-Glucose as an enriched metabolite.

**Figure S5. Coproporphyrin levels in Mabs measured by metabolomics.** Cells were grown in SCFMGlu +/- 5 µM heme. Statistical significance was measured by a two-tailed unpaired Student’s *t* test, P value calculated was **P = 0.0016.

**Figure S6**. **Metabolite levels of sulfur and methionine metabolism pathways** **A**. Levels of metabolites in Mabs cells grown in SCFMGlu (red) and SCFMGlu + 5 µM heme (blue). **B**. Simplified pathway with metabolites enriched in heme treatment colored by blue box and metabolites and pathway enzymes reduced in heme treatment colored by red box. Other measured metabolites in pathway are bolded. P-values were calculated by multiple t-test. *P values are < 0.05.

**Figure S7**. **Metabolite levels of the purine metabolism pathway** **A**. Levels of metabolites in Mabs cells grown in SCFMGlu (red) and SCFMGlu + 5 µM Heme (blue). **B**. Simplified pathway with metabolites enriched in heme treatment colored by blue box and metabolites and pathway enzymes reduced in heme treatment colored by red box. Other measured metabolites in pathway are bolded. P-values were calculated by multiple t-test. *P values are < 0.05.

**Figure S8**. **Metabolite levels of the riboflavin metabolism pathway** **A.** Levels of metabolites in Mabs cells grown in SCFMGlu (red) and SCFMGlu+ 5 µM Heme (blue). **B.** Simplified pathway with metabolites enriched in heme treatment colored by blue box and metabolites and pathway enzymes reduced in heme treatment colored by red box. . Other measured metabolites in pathway are bolded. P-values were calculated by multiple t-test. *P values are < 0.05.

**Figure S9**. **Metabolite levels of the tryptophan metabolism pathway.** **A.** Levels of metabolites in Mabs cells grown in SCFMGlu (red) and SCFMGlu+ 5 µM Heme (blue). **B.** Simplified pathway with metabolites enriched in heme treatment colored by blue box and metabolites and pathway enzymes reduced in heme treatment colored by red box. Other measured metabolites in pathway are bolded. P-values were calculated by multiple t-test. *P values are < 0.05.

**Figure S10**. **Metabolite levels of the glycerophospholipid metabolism pathway. A.**  Levels of metabolites in Mabs cells grown in SCFMGlu (red) and SCFMGlu+ 5 µM Heme (blue). **B.** Simplified pathway with metabolites enriched in heme treatment colored by blue box. P-values were calculated by multiple t-test. *P values are < 0.05.

**Figure S11. Metabolite levels UDP-Glucose and other sugars.** Levels of metabolites in Mabs cells grown in SCFMGlu (red) and SCFMGlu+ 5 µM Heme (blue). P-values were calculated by multiple t-test. *P values are < 0.05.

**Figure S12. The effect of Hb on Mabs biofilm formation. A**. Representative images of Mabs biofilm formation in SCFM with 6 µM BovHb and 3 µM Hu Hb. Arrow in HuHb well indicates HuHb aggregates **B**. The effect of heme, BovHb and human serum albumin (HSA) on Mabs unattached cell growth measured by optical density and (**C**) attached biofilm formation measured by crystal violet (CV) assay in SCFM in 96 well plates. **D**. The effect of HuHb, apo-myoglobin (Apo-Mb) and myoglobin (Mb) on Mabs unattached cell growth measured by optical density and (**E**) attached biofilm formation measured by crystal violet (CV) assay in SCFM in 96 well plates. **F.** Mabs biofilm grown for 7 days in SCFM + 3 µM HuHb. Film was stained with DNA binding dye Acridine orange (AO) to approximate total biofilm as dye binds both intracellular and extracellular DNA and cellulose binding dye calcofluor white (CW). HuHb was detected using a monoclonal antibody for human hemoglobin (Invitrogen). The merged panel includes orthogonal views along the yellow lines in the panel.

**Figure S13. The scores scatter plot of PLS-DA and OPLS-DA model as calculated and provided by Creative Proteomics.**
